# Unraveling the mechanism of ethyl acetate extract from *Prismatomeris connata* Y. Z. Ruan root in treating pulmonary fibrosis: insights from bioinformatics, network pharmacology, and experimental validation

**DOI:** 10.3389/fimmu.2023.1330055

**Published:** 2024-01-08

**Authors:** Sizheng Li, Guang Hu, Lian Kuang, Tianyu Zhou, Haiyan Jiang, Fei Pang, Jie Li, Xinyi Chen, Jie Bao, Wanfang Li, Chuangjun Li, Menglin Li, Lulu Wang, Dongming Zhang, Jinlan Zhang, Zengyan Yang, Hongtao Jin

**Affiliations:** ^1^ New Drug Safety Evaluation Center, Institute of Materia Medica, Chinese Academy of Medical Sciences and Peking Union Medical College, Beijing, China; ^2^ School of Biomedical Sciences, Hunan University, Changsha, Hunan, China; ^3^ State Key Laboratory of Bioactive Substance and Function of Natural Medicines, Institute of Materia Medica, Chinese Academy of Medical Science and Peking Union Medical College, Beijing, China; ^4^ National Medical Products Administration (NMPA) Key Laboratory of Safety Research and Evaluation of Innovative Drug, Institute of Materia Medica, Chinese Academy of Medical Sciences and Peking Union Medical College, Beijing, China; ^5^ R&D Department, Beijing Union-Genius Pharmaceutical Technology Development Co. Ltd., Beijing, China; ^6^ Section of Science & Technology, Guangxi International Zhuang Medicine Hospital, Nanning, Guangxi, China

**Keywords:** pulmonary fibrosis, macrophages polarization, ethyl acetate extract, *Prismatomeris connata* Y. Z. Ruan, inflammation/wound healing balance

## Abstract

**Introduction:**

Pulmonary fibrosis is a terminal lung disease characterized by fibroblast proliferation, extracellular matrix accumulation, inflammatory damage, and tissue structure destruction. The pathogenesis of this disease, particularly idiopathic pulmonary fibrosis (IPF), remains unknown. Macrophages play major roles in organ fibrosis diseases, including pulmonary fibrosis. The phenotype and polarization of macrophages are closely associated with pulmonary fibrosis. A new direction in research on anti-pulmonary fibrosis is focused on developing drugs that maintain the stability of the pulmonary microenvironment.

**Methods:**

We obtained gene sequencing data and clinical information for patients with IPF from the GEO datasets GSE110147, GSE15197, GSE24988, GSE31934, GSE32537, GSE35145, GSE53845, GSE49072, GSE70864, and GSE90010. We performed GO, KEGG enrichment analysis and GSEA analysis, and conducted weighted gene co-expression network analysis. In addition, we performed proteomic analysis of mouse lung tissue. To verify the results of bioinformatics analysis and proteomic analysis, mice were induced by intratracheal instillation of bleomycin (BLM), and gavaged for 14 days after modeling. Respiratory function of mice in different groups was measured. Lung tissues were retained for histopathological examination, Western Blot and real-time quantitative PCR, etc. In addition, lipopolysaccharide, interferon-γ and interleukin-4 were used to induce RAW264.7 cells for 12h in vitro to establish macrophage inflammation and polarization model. At the same time, HG2 intervention was given. The phenotype transformation and cytokine secretion of macrophages were investigated by Western Blot, RT-qPCR and flow cytometry, etc.

**Results:**

Through bioinformatics analysis and experiments involving bleomycin-induced pulmonary fibrosis in mice, we confirmed the importance of macrophage polarization in IPF. The analysis revealed that macrophage polarization in IPF involves a change in the phenotypic spectrum. Furthermore, experiments demonstrated high expression of M2-type macrophage-associated biomarkers and inducible nitric oxide synthase, thus indicating an imbalance in M1/M2 polarization of pulmonary macrophages in mice with pulmonary fibrosis.

**Discussion:**

Our investigation revealed that the ethyl acetate extract (HG2) obtained from the roots of *Prismatomeris connata* Y. Z. Ruan exhibits therapeutic efficacy against bleomycin-induced pulmonary fibrosis. HG2 modulates macrophage polarization, alterations in the TGF-β/Smad pathway, and downstream protein expression in the context of pulmonary fibrosis. On the basis of our findings, we believe that HG2 has potential as a novel traditional Chinese medicine component for treating pulmonary fibrosis.

## Introduction

Idiopathic pulmonary fibrosis (IPF), the most prevalent form of idiopathic interstitial pneumonia, manifests as a chronic, progressive, irreversible, and often lethal lung ailment. This condition is characterized by fibroblast proliferation and activation, alongside gradual accumulation of extracellular matrix in the surrounding lung tissue, thus resulting in extensive areas of abnormal pulmonary fibrosis, structural lung damage, and impaired alveolar gas exchange. Consequently, people with IPF experience a decline in respiratory capacity and ultimately succumb to respiratory failure (1–3). IPF affects predominantly men and people 60 years of age or older; the average post-diagnosis life expectancy is only 3–5 years. Currently, approximately 3 million people have IPF worldwide; the incidence rate ranges from approximately 0.09 to 1.30 per 10,000 population and continues to rise. The prevalence rate is approximately 0.33 to 4.51 per 10,000 population (4, 5). The natural progression of IPF displays heterogeneity, in that most patients exhibit a slow, progressive course, whereas others experience rapid deterioration. Disease progression is often accompanied by acute exacerbation of respiratory function, known as acute exacerbation of IPF. This phenomenon is unpredictable, and the mortality rate among people experiencing acute exacerbation can reach 50%. Currently, no effective treatment is available for the prevention and management of acute IPF exacerbation (5). The exact etiology of IPF remains unclear, although inhalation of fine particles, genetic factors, and gastroesophageal reflux are among the potential causative factors. IPF has long been considered a chronic inflammatory disease that ultimately culminates in definitive fibrosis. Examination of the lungs of patients with IPF has revealed immune cell infiltration and elevated levels of inflammatory factors. Although anti-inflammatory and immunosuppressive therapies have not achieved significant effects, concluding that inflammation does not play a role in driving IPF would be premature (6, 7).

Currently, only two oral drugs, nintedanib and pirfenidone, both FDA-approved in 2014, are used to treat IPF. However, their efficacy is limited to alleviating some symptoms and delaying disease progression. They do not significantly improve overall mortality rates, and often cause significant adverse reactions and pose financial strain on patients (8). Numerous other drugs are in various stages of clinical trials, some of which have exhibited concerning adverse reactions. Although lung transplantation is the ultimate treatment for IPF, factors such as age, financial constraints, and compatibility issues prevent most patients with IPF from undergoing this procedure (9). Human recombinant proteins, protein antagonists, specific protein antibodies, and certain combinations of drug regimens have advanced to phase II clinical studies for IPF treatment. For instance, a combination of sildenafil with pirfenidone has shown no additional therapeutic benefit beyond that of pirfenidone alone. Recombinant human serum amyloid P analog PRM-151 has shown promise in slowing lung function decline, albeit with limited effects on patients’ quality of life (10, 11). The IL-13 antibody lebrikizumab does not impede lung function decline, but has shown promise in decreasing the incidence of acute exacerbation and mortality rates in patients with IPF (12). The LPA inhibitor GLPG-1690 has shown promising results in phase II trials, but a dose-dependent increase in patient mortality risk led to early termination of the phase III trial. The LPA1 receptor blocker BMS-986020 underwent early trial termination because of its substantial adverse effects and high rate of drug discontinuation (13–15). The CTGF antibody pamrevlumab was notably found to decelerate the decline in forced crucial capacity (FVC) and acute exacerbation rates, and was subsequently investigated in phase III clinical trials (16). The GPR84 antagonist GLPG-1205 has been observed to slow FVC decline, although not statistically significantly (17). Additionally, ongoing phase II clinical trials are evaluating n-acetylcysteine, rituximab, intracellular kinase inhibitors, galectin-3 inhibitors, PDE4B inhibitors, and αvβ6/αvβ1 integrin-selective dual inhibitors (18). Overall, treatment options for patients with IPF remain limited. Thus, research and development of therapeutic drugs for IPF that effectively decelerate lung function decline; enhance patients’ quality of life; and implement more precise, targeted drug treatment regimens must critically be expedited.

Macrophages are among the most prevalent immune cell types in healthy lungs. Alveolar macrophages (AMs) make up 55% of lung immune cells and serve as the first line of defense for the lungs; these cells play crucial roles in maintaining lung equilibrium by clearing apoptotic cells and debris, regulating wound healing, and initiating immune responses against pulmonary pathogens (19). When tissue is damaged, monocytes are attracted to the alveoli and undergo differentiation into macrophages, thereby further augmenting the AM population. AMs have remarkable adaptability and can perform various functions, depending on their microenvironment. Two primary phenotypes of AM activation exist: the classical M1 type, which has pro-inflammatory roles; and the alternative M2 type, known for its anti-inflammatory properties. The transition between these two types is reversible, and is reliant primarily on cell surface markers, along with the secretion of distinct cytokines and chemokines (20). Notably, macrophage clusters, which are absent in healthy lungs, emerge in people with pulmonary fibrosis. These fibrotic AMs express genes associated with fibrosis, such as *IL1RN* and *CHI3L1*; the monocyte marker CD14; and the chemokine CCL18, produced primarily by M2 macrophages. The escalated production of chemokines leads to the recruitment of an increased number of monocyte-derived fibrocytes, which have fibroblast-like properties. Monocyte-derived AMs play a critical role in bleomycin (BLM)-induced pulmonary fibrosis, and their differentiation into AMs remains unaffected by glucocorticoids (21, 22). Additionally, M2-type macrophages facilitate myofibroblast differentiation through the production of CHI3L1 and activation of the Wnt/β-Catenin pathways (23, 24). Moreover, these cells generate various matrix metalloproteinases (MMPs), including MMP3, MMP7, and MMP8, thus further facilitating the progression of pulmonary fibrosis (25–27). Damage-associated molecular patterns and pathogen-associated molecular patterns generated during lung injury significantly influence the pulmonary fibrosis process (28, 29). Administration of alendronate, clodronate, and chlorophosphonate liposomes has shown promise in inducing apoptosis in monocytes and AMs. This approach has been found to ameliorate emphysema induced by cigarette smoke extract in mice and pulmonary fibrosis induced by BLM, as well as the effects of adenovirus vectors encoding TGF-β (30, 31). Understanding the differentiation and phenotypic transitions of monocytes/macrophages throughout the course of IPF, and investigating their effects on the progression of pulmonary fibrosis, are promising avenues for effective treatments in IPF research.

The root of the Rubiaceae plant *Prismatomeris connata* Y. Z. Ruan is believed to have pharmacological properties, including expelling phlegm, relieving asthma, and mitigating the effects of dust toxicity. HuangGen tablet, a Chinese herbal preparation, has been used in the treatment of silicosis. The primary constituents of the *Prismatomeris connata* Y. Z. Ruan root include anthraquinones, tetrahydroanthraquinones, long-chain fatty acids, alkenes (aldehydes), long-chain alkanes, organoaluminum, and polysaccharides (32, 33). Although organic aluminum and polysaccharides have been deemed effective agents in silicosis treatment, further confirmation is required (34). The alcohol extract of *Prismatomeris connata* Y. Z. Ruan root has shown promise in diminishing ALT and AST activity in experimental liver injury and fibrosis in rats, and decreasing serum HA, LN, and PCIII levels. This treatment also protects against liver injury induced by CCl4 in mice and liver fibrosis in rats (35, 36). Additionally, the ethyl acetate extract from the *Prismatomeris connata* Y. Z. Ruan root, including its component scopoletin, has anti-hepatic fibrosis effects and promotes apoptosis of hepatic astrocytes in mice (37). Our assessment of treatment of bleomycin-induced pulmonary fibrosis with the ethyl acetate extract from *Prismatomeris connata* Y. Z. Ruan root (HG2) focused on the expression of markers associated with macrophage polarization and genes/proteins associated with the TGF-β/Smad pathway in mouse lungs. We established diverse *in vitro* polarization models of RAW264.7 cells to further investigate HG2’s regulatory effects on macrophage polarization. Our findings substantiated HG2’s treatment effect on pulmonary fibrosis, and indicated that these effects may be attributable to modulation of the polarization of pulmonary macrophages. Additionally, we observed that HG2 affected the expression of TGF-β/Smad pathway members and related proteins. We propose that this previously overlooked ethyl acetate extract from *Prismatomeris connata* Y. Z. Ruan root, given its noteworthy anti-fibrosis efficacy *in vivo*, warrants consideration as a potential candidate for anti-pulmonary fibrosis treatment.

## Materials and methods

### Isolation of ethyl acetate extract HG2 from *Prismatomeris connata* Y. Z. Ruan root

A total of 60 kg of *Prismatomeris connata* Y. Z. Ruan root was subjected to extraction through reflux with 95% ethanol, with each cycle lasting 3 hours. After concentration, a yield of 1200 g of extract was achieved. This resulting extract was completely dissolved to create an aqueous solution, which was subsequently subjected to sequential extractions with petroleum ether, ethyl acetate, and n-butanol. The ethyl acetate extraction portion was subjected to decompression and concentration, thus ultimately yielding 112 g of ethyl acetate extract, denoted HG2.

### Bioinformatics analysis

#### Identification of differentially expressed genes in IPF

We obtained gene sequencing data and clinical information for patients with IPF from the GEO database. To ensure a robust sample size, we gathered data from a comprehensive set comprising 89 control lung tissue cases and 312 IPF lung tissue cases from datasets GSE110147, GSE15197, GSE24988, GSE31934, GSE32537, GSE35145, and GSE53845. Additionally, we acquired data from 68 control AMs and 27 IPF AMs from datasets GSE49072, GSE70864, and GSE90010. The data were then log_2_-transformed, and the normalize.quantiles function in the Bioconductor/R preprocessCore package was used for normalization. The common gene symbols across different datasets were extracted, and each dataset was labeled as a distinct batch. The Bioconductor/R limma package’s removeBatchEffect function was applied to mitigate any batch effects. The normalization of the pre-processed data was assessed with boxplots, and the effects of batch removal were evaluated by comparison of visual PCA plots before and after the process. Differentially expressed genes were identified with the Bioconductor/R limma package on the basis of |fold change| > 1.3 and adjusted p value < 0.05.

### Analysis of immune cell infiltration

We used the Bioconductor/R CIBERSORT package with the CIBERSORT algorithm to conduct an in-depth analysis of immune cell infiltration in lung tissue. This procedure allowed us to evaluate the abundance of 22 distinct types of immune cells in the lung tissue samples (38). Furthermore, the Bioconductor/R GSVA package and ssGSEA were used to investigate the immune infiltration specifically pertaining to AMs (39). We tailored the gene sets representative of M1 and M2 macrophages, aligning them with established biomarkers associated with the polarization of human macrophages. The M1 macrophage signature included the expression of *CD68, CD80, IL1R1, TLR2, TLR4, IL12A, IL12B, IL23A, IL27, CXCL9, CXCL10, CXCL11, CXCL16, CCL5, ARG2, NOS2, TNF, IL1A, IL1B, IL6, CD86, HLA-DMA, HLA-DMB, HLA-DOA, HLA-DOB, HLA-DPA1, HLA-DPB1, HLA-DQA1, HLA-DQA2, HLA-DQB1, HLA-DRA, HLA-DQB2, HLA-DRB1, HLA-DRB3, HLA-DRB4, HLA-DRB5, CLEC7A, STAT1, IFNG, FCGR3B, FCGR2C, FCGR1A, MERTK, and IRF5. In contrast, the M2 macrophage signature encompassed ARG1, CCL1, CCL16, CCL17, CCL18, CCL22, CCL24, CD14, CD163, CD36, CHI3L1, CHI3L2, CLEC10A, CXCL13, EBI3, F13A1, FCGR3A, IGF1, IL1R2, IL1RN, IL4R, ITGAX, MRC2, PLA2R1, RETNLB, SLAMF1, SPHK1, STAB1, TGFB1, TLR1, TLR8, VEGF, VTCN1, STAT6, IDO1, CSF1R, MSR1, CD209, CLEC4M, MS4A2, FCER1G, FCER1A, VSIG4, and IRF4* (40–43).

### Least absolute shrinkage and selection operator regression, logistic regression, and random forest analysis

The least absolute shrinkage and selection operator (LASSO) regression prediction model was constructed with the Bioconductor/R glmnet package. Parameters were set to alpha = 1 and nlambda = 1000, with lambda.min selected as the optimal lambda. Subsequently, a logistic regression model was developed with the Bioconductor/R rms package, and the calibrate function was used to fine-tune the regression model, with parameters B = 1000 and sls = 0.05.

For random forest analysis, the Bioconductor/R pacman package was used, with the randomForest function. The mtry node value was determined on the basis of the minimum error, whereas the ntree value was selected according to image stability trends.

To identify the top ten critical differential immune cells, we used the mean decrease accuracy and mean decrease Gini metrics. Further refinement of the key differential immune cells was achieved through a comparative assessment across the three algorithms.

### GO, KEGG, and CTpathway analyses

We performed GO and KEGG enrichment analyses with the Bioconductor/R clusterProfiler, enrichment, and ggplot2 packages. Additionally, for CTpathway enrichment analysis, we used the online analysis platform available at http://www.jianglab.cn/CTpathway/ (44).

### Gene set enrichment analysis

The gene set enrichment analysis (GSEA) analysis of patients with IPF was conducted with the gseGO and gseKEGG functions in the Bioconductor/R clusterProfiler package. Results meeting the criteria of an |normalized enrichment score| > 1, adjusted p value < 0.05, and false discovery rate (FDR) < 0.25 were considered statistically significant.

### Weighted gene co-expression network analysis

We used the Bioconductor/R weighted gene co-expression network analysis (WGCNA) package to construct co-expression networks between IPF lung tissue and normal samples, as well as between IPF AMs and normal samples (45). The sample’s phenotype was categorized as either IPF group or normal control. For lung tissue, the soft threshold was set to β = 4, thus yielding a scale-free R^2^ of 0.85. For AMs, the soft threshold was adjusted to β = 9, and a scale-free R^2^ of 0.85 was maintained. Subsequently, the co-expression matrix was generated with the expression matrix, and the respective β values were determined. Genes exhibiting similar expression patterns were grouped into corresponding gene modules, thus establishing co-expression modules (**Supplementary Figures S2, S3**). Characteristic gene functions were applied to assess disparities in modular characteristic genes (Mes) and the correlation between modules and phenotypes. Pearson coefficients were computed to determine the correlation between genes and phenotypes within modules, thereby identifying central hub genes.

### Network pharmacology analysis

#### Collection of candidate ingredients and potential drug targets

The compounds with established structures found in the root of *Prismatomeris connata* Y. Z. Ruan were identified through a comprehensive literature review. These compounds’ structural formulas were rendered with ChemDraw software (CambridgeSoft, USA) and subsequently transcribed into Simplified Molecular Input Line Entry System (SMILES) notation. With the SwissADME online platform (http://www.swissadme.ch/), we systematically screened the components to identify active compounds. The screening criteria were as follows: oral bioavailability ≥ 30%; “high” gastrointestinal absorption; and affirmative outcomes for the Lipinski, Ghose, Veber, Egan, and Muegge criteria. Subsequently, we sought to predict the potential targets of these active compounds by using the online databases SwissTargetPrediction (http://www.swisstargetprediction.ch/), SuperPred (https://prediction.charite.de/), and SEA (https://sea.bkslab.org/). The outcomes from these three databases were amalgamated, and any duplicate targets were removed, thus yielding potential targets for the identified candidate components.

#### Collection of therapeutic targets for IPF

IPF therapeutic targets were curated from GEO and various online disease databases. These targets included differentially expressed genes, hub genes enriched via CTpathway, and hub gene modules acquired through WGCNA. Specifically, for lung tissue sample data, we focused on the blue and turquoise modules, whereas for AM sample data, we focused on the purple and light green modules. After removal of any duplicate genes, therapeutic targets were subsequently extracted from GEO.

Using “idiopathic pulmonary fibrosis” as the critical keyword, we gathered comprehensive therapeutic target data associated with IPF from three online databases: GeneCards (https://www.genecards.org/), DisGeNET (https://www.disgenet.org/), and OMIM (https://omim.org/). Subsequently, using Cytoscape 3.9.2 software, we integrated the amassed targets and constructed a robust disease-target network.

#### Construction of the protein-protein interaction network

Using the String online database (https://string-db.org/) for the protein interaction network analysis, we specified “Homo sapiens” in the species column and selected “confidence” in the meaning of network edges column. To enhance specificity, we excluded free proteins, focusing solely on relevant data between targets. Subsequently, we imported the obtained data into Cytoscape 3.9.2 software, with the CytoNCA plug-in, to screen for core targets. The protein-protein interaction (PPI) network was constructed according to the criteria of closeness ≥ 0.001899, focus betweenness ≥ 278.766, and degree ≥ 37.4789.

### Proteomics detection and analysis

#### Protein extraction

Each sample to be lysed was treated with lysis buffer comprising 1% SDS, 8 M urea, and 1× protease inhibitor cocktail (Roche Ltd. Basel, Switzerland). After vortex mixing, the sample underwent three rounds of vigorous shaking and grinding with a high-flux tissue grinder. Subsequently, the sample was lysed on ice for 30 minutes with gentle mixing once every 10 minutes. Afterward, the lysate was subjected to centrifugation for 20 minutes at 12,000 g and 4°C. The supernatant was carefully extracted, and the protein concentration was then determined with a quantitative BCA protein kit.

#### Proteolysis

From each sample, 100 μg of protein was carefully transferred into a fresh EP tube and then adjusted to a constant volume of 100 μL with 8 M urea. Next, 2 μL 0.5 M TCEP was added, and the mixture was incubated for 1 hour at 37°C. Subsequently, 4 μL of 1 M iodoacetamide was added, and precautions were taken to avoid exposure to light; the mixture was allowed to stand at room temperature for 40 minutes.

Subsequently, acetone, pre-chilled to −20°C, was added to the sample at a ratio of 1:5 (sample to acetone, volume ratio), and the solution was allowed to precipitate overnight at −20°C. The supernatant was carefully discarded after high-speed centrifugation for 20 minutes at 12,000 g and 4°C.

Next, 1 mL of 90% acetone solution, also pre-cooled at −20°C, was added, and the sample was subjected to the cleaning process again through vigorous vortex mixing. The supernatant was again discarded after high-speed centrifugation for 20 minutes at 12,000 g and 4°C. This cleaning process was repeated twice. The sample was then allowed to air dry at room temperature until the acetone precipitation surface had completely evaporated.

The dried sample was then reconstituted in 100 μL of 100 mM TEAB with a mass ratio of 1:50 (enzyme to protein). Trypsin (Promega, Madison, WI) was added, and the sample was subjected to overnight hydrolysis at 37°C. After desalting with a C18 desalting column, the peptide concentration was determined, and the solution was subsequently freeze-dried.

#### Nano-HPLC-MS/MS data acquisition

A suspension was prepared by addition of 30 μL of solvent A (A: 0.1% formic acid aqueous solution) to each sample. Subsequently, 9 μL of this suspension was combined with 1 μL of 10× iRT peptide. The resulting mixture underwent separation via nano-LC followed by analysis through online electrospray tandem mass spectrometry. The entire system comprised an Orbitrap Fusion™ Lumos™ Tribrid™ Mass Spectrometer (Thermo Fisher Scientific, USA) in series with an EASY-nano LC 1200. A total of 6 μL of sample, with an analytical column (Acclaim PepMap C18, 75 μm × 25 cm) was subjected to a 90-minute gradient separation. The column flow rate was precisely controlled at 250 nL/min, with a column temperature maintained at 40°C. Additionally, an electrospray voltage of 2 kV was used, and the chromatographic gradient spanned 120 minutes. Phase A comprised a 0.1% formic acid aqueous solution, whereas phase B consisted of an ACN solution containing 0.1% formic acid.

The mass spectrometer was operated in data-independent acquisition mode with the following parameters: (1) MS1 scanning range (m/z): 350–1200; resolution: 120,000; AGC target: 1×10^6^; maximum injection time: 100 ms; (2) HCD-MS/MS: resolution: 50,000; AGC target: 1×10^5^; maximum injection time: 86 ms; collision energy: 33; and (3) variable window acquisition, with 60 windows per set, each overlapping by 1 m/z.

#### Data analysis

The DIA data underwent analysis with the default parameters of Spectronaut14 (BGS Factory Settings). The retention time and quality window were automatically adjusted in iRT peptide software to ascertain the optimal extraction window. For protein qualification, the criteria applied were a precursor threshold of 1.0% FDR and a protein threshold of 1.0% FDR. A decoy database was generated with a mutation strategy, akin to disrupting a random number of amino acid sequences (at least 2 amino acids and half the total length of the maximum peptide).

Spectronaut automatically performed corrections by using all MS1 spectra that met the screening criteria to calculate expression levels, while excluding all interfering fragment ions, unless fewer than three fragment ions were present. The quantification of protein groups relied on the average peak area of the first three peptides, ensuring a threshold of less than 1.0% FDR.

For differential screening, proteins were selected on the basis of Welch’s ANOVA test analysis, with criteria set at p-value < 0.05 and |fold change| > 1.5.

### Animal treatment and determination of respiratory function

Specific pathogen-free (SPF) male C57BL/6N mice, weighing between 18 and 22 g were procured from Beijing Vital River Laboratory Animal Technology Co., Ltd. The company holds a production license (SCXK- (Beijing) 2016-0011) issued by the Beijing Science and Technology Commission. The animals were housed in the facilities of Beijing Union-Genius Pharmaceutical Technology Development Co., Ltd. This company is further accredited by the Association for Assessment and Accreditation of Laboratory Animal Care (AAALAC International) under number 143.

Each cage housed five mice, and a controlled environment was provided with a room temperature of (25 ± 2)°C and 45% relative humidity. A 12-hour light-dark cycle was maintained, and the mice were given unrestricted access to food and water throughout the feeding period. The experimental protocol was approved by the company’s Experimental Animal Welfare Ethics Committee. The experimental animal license number is SCXK (Beijing) 2020-0021, and the experimental animal certificate number is 2018-002 (research).

A total of 65 SPF male C57BL/6N mice, 6–8 weeks of age, weighing 18–22 g, were selected. Among them, ten mice served as the control group, whereas the remaining 55 mice were subjected to BLM treatment for inducing pulmonary fibrosis. After 1 week of acclimation, each mouse underwent a precise intratracheal injection of either BLM (1.25 mg/kg, dissolved in sterile PBS buffer, provided by Zhejiang HISUN Pharmaceutical Co., LTD., China) or sterile PBS buffer (50 μL) with an HRH-MAG4 quantitative pulmonary fluid atomizer from Beijing HuiRongHe Technology Co., Ltd. (Beijing, China).

On the 15th day after BLM treatment, HG2 (at doses of 100 mg/kg, 200 mg/kg, or 400 mg/kg), nintedanib (50 mg/kg, sourced from the Institute of Materia Medica, Chinese Academy of Medical Sciences, China), or vehicle (0.5% CMC-Na) was administered via gastric gavage once daily for 14 consecutive days. Within 24 hours after the last administration, three mice were randomly selected from each group and humanely euthanized, and the residual blood in the lung tissue was cleared through cardiac perfusion. The lungs were then extracted; the left lung was subjected to histopathological examination, and the right lung was promptly frozen at −80°C.

The remaining mice were anesthetized via intraperitoneal injection of pentobarbital sodium (90 mg/kg), then underwent tracheotomy and tracheal intubation, and were connected to an Flexivent small animal pulmonary function instrument from SCIREQ (city, France). Various pulmonary function parameters, including respiratory resistance (Rrs), central airway resistance (Crs), peak expiratory flow (PEF), FVC, and forced expiratory volume in the first 0.1 second (FEV0.1), were measured.

Bronchoalveolar lavage was performed with pre-chilled PBS buffer at 4°C (1 ml/each, performed three times), and the bronchoalveolar lavage fluid (BALF) was collected and stored at 4°C for subsequent cytokine analysis.

To further investigate the mechanism of HG2, we selected 30 SPF male C57BL/6N mice, 6–8 weeks old, with an average body weight of 18–22 g. Among these, 25 mice were subjected to induced pulmonary fibrosis with the previously described modeling approach, whereas five mice were designated as the control group.

On the 15th day after administration of BLM, HG2 was administered via gastric gavage at varying doses (50 mg/kg, 100 mg/kg, and 200 mg/kg), alongside nintedanib (at a dose of 50 mg/kg), or a vehicle solution (0.5% CMC-Na), once per day for 14 days. Subsequently, all mice were euthanized within 24 hours of the final administration. Residual blood in the lung tissue was removed via cardiac perfusion. The lungs were then extracted, promptly frozen at −80°C, and prepared for subsequent western blot analysis.

### Histological evaluation

The left lung samples from the mice were preserved in 4% paraformaldehyde for more than 72 hours. Subsequently, the samples underwent dehydration and paraffin embedding, then were sectioned into slices with 4 μm thickness. These slices were then subjected to staining with hematoxylin and eosin, as well as Masson’s trichrome, both from Beijing SoonBio Technology Co., Ltd., China. Subsequently, the samples were imaged with a NIKONCI-S microscope (Nikon, Japan) in conjunction with a DS-F12 imaging system (Nikon).

### Cell culture and determination of cell viability

RAW264.7 cells, generously provided by the Stem Cell Bank at the Chinese Academy of Sciences, were cultivated in DMEM:F-12 1:1 medium (C3132-0500, Viva Cell, Israel) supplemented with 10% fetal bovine serum (C04001-500, Viva Cell, Israel), 100 U/mL penicillin, 0.1 g/mL streptomycin, and 12.5 U/mL mycostatin (C3422-0100, Viva Cell, Israel) under 5% CO_2_ at 37°C until they reached 70% confluence.

At 24 hours after RAW264.7 cell seeding in 96-well cell culture plates, the medium was aspirated, and 200 μL of either vehicle (0.5% DMSO) or various concentrations of HG2 solution (673.72, 336.86, 168.43, 84.2, 42.10, 21.05, and 10.50 μg/mL) was added to each well. The plates were then incubated for 12 hours at 37°C under 5% CO_2_. Subsequently, the medium was removed, and the cells were treated with Cell Counting Kit-8 (CCK-8) reagent (10 μL, CK001-500T, Lablead, China) for 1.5 hours at 37°C. The optical density (OD) at 450 nm was measured with a TriStar2 SLB942 fluorescence enzyme labeling instrument (Berthold Technologies, Germany). Cell viability was calculated with the following formula: cell viability (%) = [OD (treatment) − OD (blank)]/[OD (control) − OD (blank)].

After determination of HG2’s median lethal concentration (LC50) in RAW264.7 cells, the cells were subjected to the following treatments: serum-free medium, serum-free medium with 1 μg/mL lipopolysaccharide (LPS), or 1 μg/mL LPS combined with 3 ng/mL interferon (IFN)-γ or 20 ng/mL IL-4. Additionally, cells were treated with serum-free medium containing 1 μg/mL LPS or 1 μg/mL LPS combined with 3 ng/mL IFN-γ or 20 ng/mL IL-4, along with varying concentrations of HG2 (84.2, 42.1, 21.05, and 10.5 μg/mL). Subsequently, cells and supernatants were collected for nitric oxide (NO) and cytokine determination, western blotting, real-time quantitative PCR (RT-qPCR), flow cytometry, and immunocytochemistry.

### Western blotting, immunohistochemistry, multiple fluorescence immunohistochemistry, and immunocytochemistry

RAW264.7 cells were harvested, and 200 μL of RIPA lysis buffer was added to each cell sample. The cells were thoroughly lysed through a series of freeze-thaw cycles. Subsequently, the lysates were centrifuged for 20 minutes at 12,000 rpm and 4°C and, and the resulting supernatant was collected to obtain intracellular proteins. For extracellular protein extraction, culture supernatants were collected and mixed with pre-cooled acetone at a 1:4 volume ratio. The mixture was then frozen at −20°C for a minimum of 12 hours to ensure complete protein precipitation. Afterward, the sample was centrifuged for 20 minutes at 12,000 rpm and 4°C, and the resulting pellet was collected. This pellet contained the extracellular proteins, which were reconstituted in the previously obtained intracellular protein solution to yield the total cellular protein.

Total protein was extracted from lung tissues with an animal tissue total protein extraction kit (BC3790, Solarbio, China). A BCA protein quantification kit (B5000–500T, Lablead, China) was used to determine protein concentrations. Cell samples (50 μg) and tissue samples (70 μg) were separated on 4%–12% SDS-PAGE gels and then transferred to a PVDF membrane. The membrane was blocked with 5% skim milk at room temperature for 1 hour, then incubated overnight with primary antibodies (1:1500) at 4°C. After thorough washing, the membrane was incubated with HRP-conjugated secondary antibodies (1:3000) for 1–2 hours at room temperature. Details regarding the primary and secondary antibodies are provided in the **Supplementary Materials** (**Supplementary Table S1**). After additional washes, protein signals were detected with an ECL kit (E1060, Lablead, China). Quantitative analysis was conducted with ImageJ (National Institute of Mental Health, USA).

The aforementioned tissue sections underwent immunohistochemical staining for collagen I (1:400, ab279711, Abcam, UK) after high-pressure heat-induced antigen retrieval, with a SABC-AP immunohistochemical staining kit (SA1052, Boster, China). Images were captured with an AxioVert. A1 Microscopic Imaging System (CARL ZEISS, Germany) and subjected to quantitative analysis in ImageJ.

Multiple fluorescence immunohistochemical staining was performed with a PANO 4-plex IHC kit (10001100100, Panovue, China). The aforementioned tissue slices were subjected to high-pressure heat-induced antigen retrieval and sealed with a specialized immunohistochemistry sealing solution. Subsequently, the corresponding primary antibody (1:400) and HRP-labeled secondary antibody were sequentially applied. After TSA signal amplification, the slices underwent high-pressure heat treatment after each TSA application to decrease background noise. After the labeling of all antigens, nuclei were stained with DAPI, and images were captured with an AxioVert. A1 microscopic imaging system. ZEN3.1 (CARL ZEISS, Germany) was used for image processing and Plot Profile analysis, whereas ImageJ was used for quantitative analysis.

RAW264.7 cells were seeded onto cell tablets (BS-24-RC, Bio-sharp, China). After cell adhesion, the cells were fixed with 4% paraformaldehyde, permeabilized with 0.1% Triton, and blocked with 10% goat serum (50012-8615, SiJiQing, China). Subsequently, the cells were incubated with the corresponding primary antibody (1:500) and fluorescently labeled secondary antibodies (1:500). Nuclei were counterstained with DAPI (SIGMA-ALDRICH, Germany), and images were visualized with an AxioVert. A1 microimaging system. The images were further processed with ZEN3.1.

### Determination of nitric oxide and cytokines

The supernatants from cell cultures and mouse BALF were carefully collected, subjected to centrifugation for 10 minutes at 1000 rpm and 4°C, and the supernatant subsequently aspirated. The NO content within the cell culture supernatant was quantified with an NO one-step assay kit (A013-2-1, Nanjing Jiancheng Bioengineering Institute, China). Additionally, the expression levels of key cytokines, including IL-6, IL-10, IL-12, tumor necrosis factor (TNF)-α, and TGF-β1 in the cell culture supernatant, were determined according to the protocols outlined by the respective commercial enzyme-linked immunosorbent assay (ELISA) kits (Boster, China).

Furthermore, the levels of cyclooxygenase (COX)-2, plasminogen activator inhibitor (PAI)-1, MMP7, and IL-17B in BALF were evaluated with respective commercial ELISA kits (all from Cloud-clone, China). Finally, the levels of TGF-β1, IL-10, E-cadherin, IL-17A, and MMP3 in BALF were determined with respective commercial ELISA kits (all from Boster) according to the manufacturer’s instructions for accurate determination.

### Real-time quantitative PCR analysis

Total RNA was extracted from both RAW264.7 cells and lung tissue homogenate with an RNeasy™ Plus animal RNA extraction kit (R0032, Beyotime, China). Subsequently, a PrimeScript™ RT reagent Kit with gDNA Eraser (Perfect Real Time) (RR047A, Takara, Japan) was used for cDNA reverse transcription. The quantitative PCR reactions were conducted in a 20 μL reaction mixture system using TB Green Premix Ex TaqTM II (Tli RNaseH Plus) (RR820A, Takara, Japan) and specific primers. Amplification was performed with an ABI 7900HT 96 real-time PCR system (Applied Biosystems, USA). The mRNA levels were normalized to those of GAPDH, and the fold change in mRNA was calculated with the ΔΔCt method. Primer sequences are provided in the **Supplementary Material** (**Supplementary Table S2**).

### Flow cytometry

RAW264.7 cultured cells were harvested by centrifugation at 3000 rpm at 4°C for 5 minutes and subsequently labeled with anti-NOS2-PE (696805, Biolegend, USA), anti-MHC-II-FITC (107606, Biolegend, USA), and anti-F4/80-APC (123116, Biolegend, USA). Flow cytometry was performed with a FACS Verse™ Cell Analyzer (BD Biosciences, USA), and the data were analyzed in FlowJo software (Tree Star, USA) to determine the percentage macrophage polarization (iNOS^+^F4/80^+^ or MHC-II^+^F4/80^+^).

### Statistical analysis

The data are presented as mean ± standard deviation (SD), and each experiment was conducted independently and repeated at least three times. Initially, the homogeneity of variances was assessed. In cases of homogeneous variances, one-way ANOVA was used to determine the statistical significance of differences between various samples (P < 0.05). In the event of heterogeneous variances, an independent samples Student’s t-test was used to assess whether the differences between samples were statistically significant (P < 0.05). Data analysis was performed in IBM SPSS Statistics 21.0 (IBM, USA), and GraphPad Prism 8.0.2 (GraphPad Software, USA) was used for graphical representation.

## Results

### Bioinformatics analysis reveals a significant biological role of macrophage polarization in idiopathic pulmonary fibrosis

**Figure 1:**
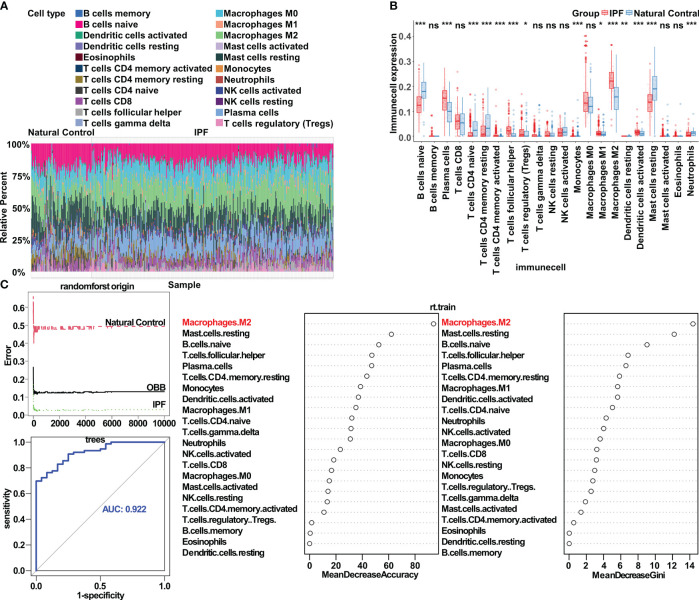
Bioinformatics analysis highlights the crucial role of macrophage polarization in idiopathic pulmonary fibrosis. **(A)** Immune cell infiltration in both IPF lung tissue and control tissue. **(B)** Significantly different infiltrates of immune cells in IPF lung tissue and control tissue, assessed with the Wilcoxon test; ^*^p < 0.05; ^**^p< 0.01; ^***^p < 0.001. **(C)** Application of the random forest algorithm, respectively, in analysis of the distinct infiltrates of immune cells in IPF lung tissue and control tissue. ns, no significance.

To investigate immune cell infiltration patterns in the lungs of patients with IPF versus healthy people, we analyzed the expression matrix (comprising seven datasets) with Cibersort. Our findings indicated a notable increase in the infiltration levels of plasma cells, T follicular helper cells, M2 macrophages, and activated dendritic cells in patients with IPF compared with healthy controls. In contrast, the infiltration levels of naive B cells, naive CD4 T cells, resting CD4 memory T cells, resting mast cells, and neutrophils exhibited a significant decrease. Additionally, a slight increase was observed in the infiltration levels of M1 macrophages and regulatory T cells (Tregs) (**Figures 1A, B**).

Further exploration of the correlation between IPF and immune cell infiltration in the lungs revealed a strong association between M2 macrophages and IPF. These M2 macrophages exhibited a positive correlation with activated dendritic cells and a negative correlation with naive B cells (**Supplementary Figure S1A**). With LASSO regression, we identified ten immune cell types closely associated with IPF (**Supplementary Figure S1B**). Subsequently, using multivariate logistic regression to control for potential confounding factors, we determined that M2 macrophages, plasma cells, T follicular helper cells, and monocytes were characteristic immune cell types in IPF (**Supplementary Figure S1C**). In the random forest algorithm, immune cell types were ranked on the basis of the feature weights of the mean decrease accuracy and mean decrease Gini, and the top ten cell types were selected as key contributors (**Figure 1C**). By combining the results from the three algorithms, we conclusively established M2 macrophages as the most critical immune cell type in IPF. Additionally, T follicular helper cells and plasma cells also played substantial roles in the condition. To corroborate our findings, we assessed the gene expression of markers associated with macrophage polarization in the lung tissue in patients with IPF compared with healthy controls, and observed the anticipated expression patterns (**Supplementary Figure S1D**).

**Figure 2:**
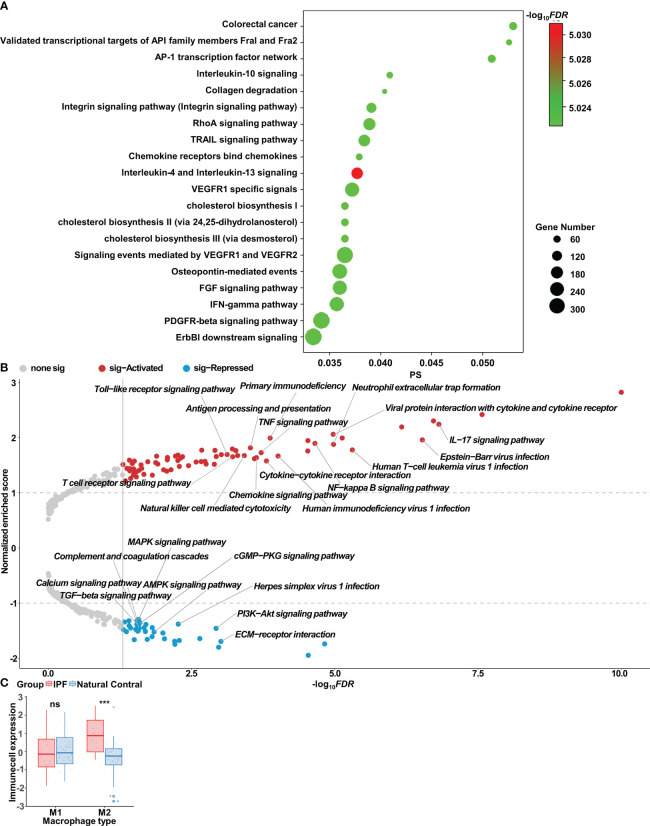
Bioinformatics analysis of alveolar macrophages in idiopathic pulmonary fibrosis. **(A)** Enrichment analysis of gene expression, determined with CTpathway. **(B)** Volcano plot of gene set enrichment analysis (GSEA) in IPF alveolar macrophages compared with control alveolar macrophages, determined with gseKEGG. **(C)** Wilcoxon test results indicating significant differences in infiltrates of immune cells in IPF alveolar macrophages compared with control alveolar macrophages; ns, no significance; ^***^p < 0.001.

We further assessed differences in AMs between patients with IPF and healthy individuals, which exhibited elevated expression of genes associated with the inflammatory response and macrophage polarization, including CCL2, CXCL3, CD36, and TLR2 (**Supplementary Figure S2A**). CTpathway-based enrichment analysis of gene expression revealed pathways associated predominantly with macrophage polarization and collagen, such as the IL-4, IL-13, IL-10, and IFN-γ pathways; MHC II antigen presentation; and collagen degradation (**Figure 2A**, **Supplementary Figure S2B**). GSEA analysis further demonstrated the activation of immune-associated KEGG pathways (e.g., Toll-like receptor signaling pathway, TNF signaling pathway, and NF-κB signaling pathway) in the AMs of patients with IPF (**Figure 2B**). Notably, inflammatory, immune, and cytokine-associated GO pathways, including immune response-activated cell surface receptor signaling pathway, viral response, and lymphocyte activation regulation, exhibited substantial alterations (**Supplementary Figure S2C**). Through ssGSEA analysis of the AM dataset, we observed significantly greater abundance of M2 AM infiltration in patients with IPF than healthy controls (**Figure 2C**).

### Network pharmacology analysis elucidates the potential therapeutic targets and pathways of *Prismatomeris connata* Y. Z. Ruan root in idiopathic pulmonary fibrosis

Traditional Chinese medicine is distinguished by its multi-component and multi-target synergy. Through an extensive literature review, we identified 51 compounds with known structures in the root of *Prismatomeris connata* Y. Z. Ruan (**Supplementary Figure S3A**). SwissADME (http://www.swissadme.ch/) was used to screen for active ingredients, thus yielding 36 compounds (compounds 1–36). Furthermore, SwissTargetPrediction (http://www.swisstargetprediction.ch/), SuperPred (https://prediction.charite.de/), and SEA (https://sea.bkslab.org/) were used to predict potential drug targets for these active compounds, thus yielding a comprehensive list of 670 target genes.

For a deeper exploration of the targets of active compounds in IPF, we curated therapeutic target information for IPF from various datasets in the GEO database, including lung tissue samples from GSE110147, GSE15197, GSE24988, GSE31934, GSE32537, GSE35145, and GSE53845, as well as AM samples from GSE49072, GSE70864, and GSE90010. Differentially expressed genes were identified with the Bioconductor/R limma package. Disease-associated gene modules were examined via WGCNA, and hub genes were extracted through CTpathway analysis.

**Figure 3:**
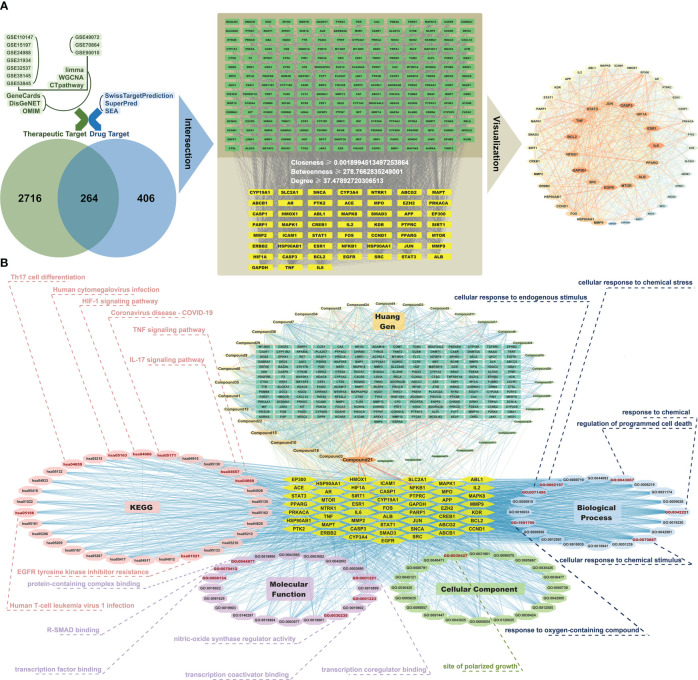
Network pharmacology analysis reveals potential therapeutic targets and pathways of *Prismatomeris connata* Y. Z. Ruan root in idiopathic pulmonary fibrosis. **(A)** Pharmacological targets of HG2 in idiopathic pulmonary fibrosis (IPF). **(B)** Component-target-pathway network of HG2 in IPF.

Simultaneously, therapeutic target information for IPF was obtained from three online databases: GeneCards (https://www.genecards.org/), DisGeNET (https://www.disgenet.org/), and OMIM (https://omim.org/), thus resulting in 2980 targets (**Figure 3A**). With the CytoNCA plug-in in Cytoscape software, we filtered core targets with criteria including closeness ≥ 0.001899, betweenness ≥ 278.766, and degree ≥ 37.4789, thus resulting in the identification of 52 core targets (**Figure 3A**).

The outcomes of GO enrichment and KEGG enrichment analyses indicated that the active ingredients within the root of *Prismatomeris connata* Y. Z. Ruan regulate pathways associated primarily with immunity, such as the TNF signaling pathway, IL-17 signaling pathway, pathways associated with the coronavirus disease COVID-19, and pathways associated with cell differentiation, such as Th17 cell differentiation and sites of polarized growth in IPF therapy (**Figure 3B**).

### Identification of potential pathogenesis and treatment mechanisms of HG2 in idiopathic pulmonary fibrosis through proteomic strategies

**Figure 4:**
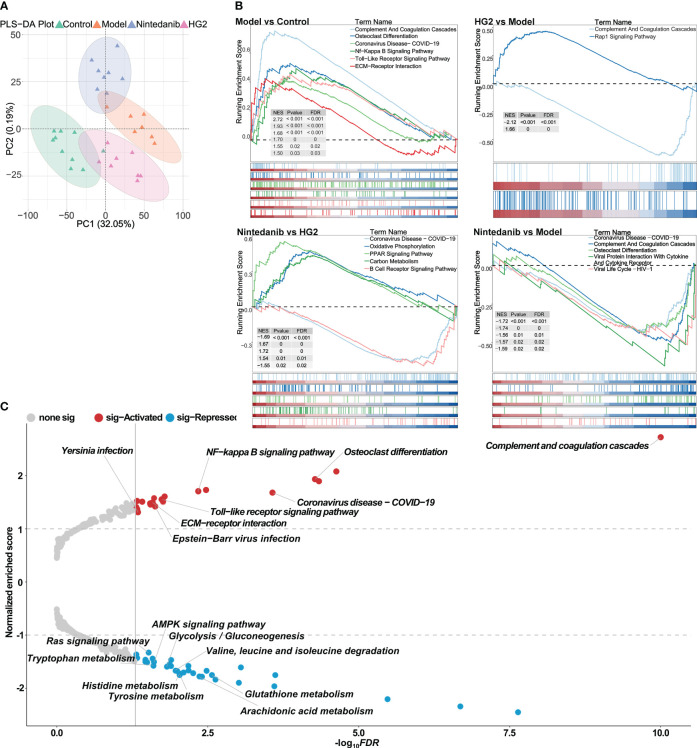
Proteomic strategies uncover the potential pathogenesis of idiopathic pulmonary fibrosis and the mechanism of HG2. **(A)** Partial least squares discriminant analysis of the control, model, nintedanib, and HG2 groups. **(B)** GSEA analysis in BLM-induced lung fibrosis tissue compared with control tissue, determined with gseKEGG. **(C)** Volcano plot of GSEA analysis in BLM-induced lung fibrosis tissue compared with control tissue, determined with gseKEGG.

A data independent model was used for proteomic analysis across all sample groups. By setting the FDR of the peptides to 1% and ensuring that each protein was represented by at least one characteristic peptide, we identified 6035 proteins in all four sample groups. PLS-DA revealed clear distinctions between each group, most prominently between the model group and control group. Furthermore, after administration of HG2 or nintedanib, we observed a discernible trend in reversion of protein expression levels (**Figure 4A**).

Further GSEA demonstrated significant activation of immune-associated pathways, such as the complement and coagulation cascade, NF-κB signaling pathway, Toll-like receptor signaling pathway, and pathways associated with COVID-19, as well as collagen-associated pathways including ECM receptor interaction, in the model group compared with the control group (**Figure 4C**). These findings were consistent with the results obtained from the bioinformatics analysis. Additionally, pathways associated with immunoglobulin production, innate immune response, inflammatory response, and viral response were notably activated in the model group compared with the control group (**Supplementary Figure S4A**).

HG2 and nintedanib exhibited distinct action profiles. Whereas both compounds down-regulated expression of the members of complement and coagulation cascade, HG2 demonstrated more potent inhibition of pathways associated with immunoglobulin production, including molecular regulator production, immunoglobulin production, humoral immune response, and complement activation, in the immune response (**Figure 4B**, **Supplementary Figure S4A**). In comparison to the HG2 group, the nintedanib group displayed significant up-regulation of the expression of Coronavirus disease-COVID19, oxidative phosphorylation, and PPAR signaling pathway members, whereas the expression of carbon metabolism and B-cell receptor signaling pathway members was notably down-regulated. These findings indicated that HG2 exerts a more focused role in immune regulation than nintedanib (**Figure 4B**, **Supplementary Figure S4A**).

### HG2 inhibits cytokine expression and macrophage polarization *in vitro*

**Figure 5:**
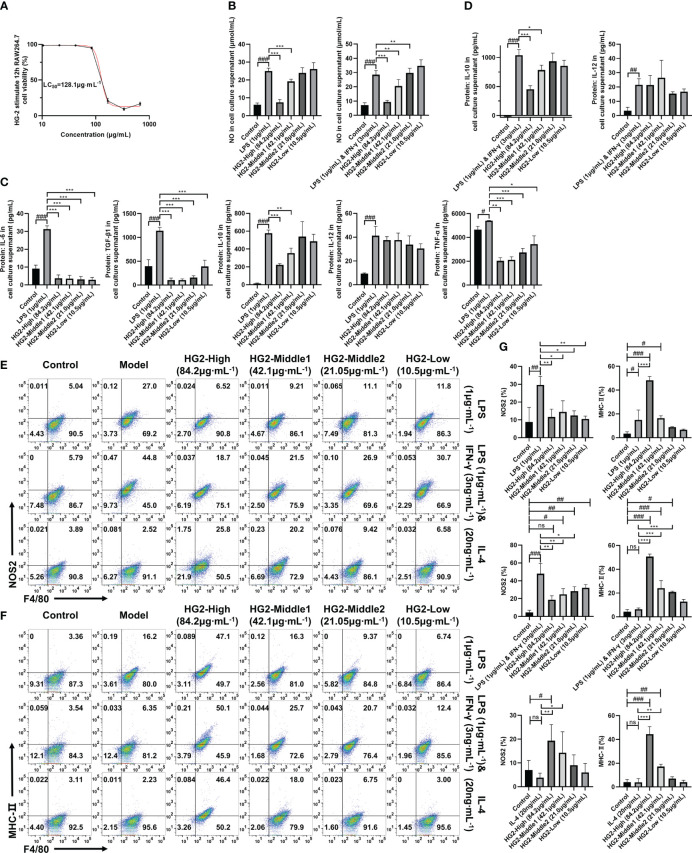
HG2 suppresses the expression of cytokines and polarization-associated biomarkers in macrophages *in vitro*. **(A)** Survival rate of RAW264.7 cells stimulated with different concentrations of HG2 for 12 hours, measured with the CCK-8 method. **(B)** NO content determined in the supernatant of RAW264.7 cells after stimulation with LPS (1 μg/ml) or LPS (1 μg/mL) combined with IFN-γ (3 ng/mL) and HG2 treatment. **(C)** Content of IL-6, TGF-β1, TNF-α, IL-10, and IL-12 in the supernatant of RAW264.7 cells, determined by ELISA after stimulation with LPS (1 μg/ml) and HG2 treatment. **(D)** Content of IL-10 and IL-12 in the supernatant of RAW264.7 cells, determined by ELISA after stimulation with LPS (1 μg/mL) combined with IFN-γ (3 ng/mL) and HG2 treatment. **(E–G)** Expression of iNOS and MHC-II in RAW264.7 cells, measured by flow cytometry after stimulation with LPS (1 μg/mL) or LPS (1 μg/mL) combined with IFN-γ (3 ng/mL) or IL-4 (20 ng/mL) and HG2 treatment. Statistical values are expressed as mean ± SD, and each experiment was independently repeated three times. Compared with the control group, ^###^p < 0.001, ^##^p < 0.01, ^#^p < 0.05; compared with the model group, ^*^p < 0.05, ^**^p < 0.01, ^***^p < 0.001. ns, no significance.

We further examined HG2’s effects on cytokine release and macrophage polarization by using an *in vitro* macrophage model. Initially, we assessed the effect of HG2 stimulation at varying concentrations on RAW264.7 cell proliferation over 12 hours. The calculated 12-hour LC50 of HG2 on RAW264.7 cells was 128.1 ± 1.4 μg/mL (**Figure 5A**). On the basis of this result, we established four concentrations (84.2, 42.1, 21.05, and 10.5 μg/mL) as distinct HG2 administration groups.

Next, RAW264.7 cells were stimulated with LPS (1 μg/mL) for 12 hours and subsequently treated with different concentrations of HG2. We determined cytokine and NO levels in the supernatant with ELISA and NO one-step assay kits. HG2 significantly decreased the levels of IL-6, TGF-β1, TNF-α, IL-10, and NO in the supernatant of RAW264.7 cells induced by LPS. Notably, the decrease in TGF-β1 and TNF-α exhibited a dose-dependent pattern. We also observed a similar trend when HG2 was applied to RAW264.7 cells co-stimulated with LPS (1 μg/mL) and IFN-γ (3 ng/mL), thus substantially decreasing NO and IL-10 release (**Figures 5B–D**).

**Figure 6:**
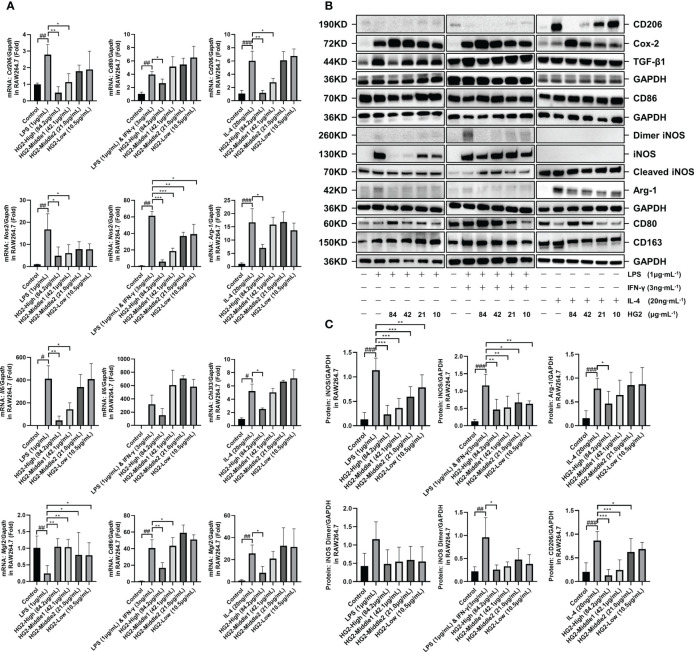
HG2 suppresses the expression of polarization-associated biomarkers in macrophages *in vitro*. **(A)** mRNA expression of polarization-associated markers in RAW264.7 cells, determined by RT-qPCR under stimulation with LPS (1 μg/mL) or LPS (1 μg/mL) combined with IFN-γ (3 ng/mL) or IL-4 (20 ng/mL) and HG2 treatment. **(B, C)** Expression of CD206, CD86, iNOS, Arg-1, CD80, and CD163 in RAW264.7 cells, determined with western blotting after stimulation with LPS (1 μg/mL) or LPS (1 μg/mL) combined with IFN-γ (3 ng/mL) or IL-4 (20 ng/mL) and HG2 treatment, as well as the expression of total TGF-β1 and COX-2. Statistical values are expressed as mean ± SD, and each experiment was independently repeated three times. Compared with the control group, ^###^p < 0.001, ^##^p < 0.01, ^#^p < 0.05; compared with the model group, ^*^p < 0.05, ^**^p < 0.01, ^***^p < 0.001.

To understand HG2’s influence on macrophage polarization, we subjected RAW264.7 cells to stimulation with LPS (1 μg/mL) separately, LPS (1 μg/mL) combined with IFN-γ (3 ng/mL) and IL-4 (20 ng/mL) separately for 12 hours, with subsequent HG2 treatment. We monitored changes in mRNA (**Figure 6A**, **Supplementary Figure S5A**) and protein (**Figures 6B, C**) expression levels of polarization-associated markers in RAW264.7 cells. HG2 notably suppressed the elevation in *Cd206*, *Nos2*, and *Il6* mRNA expression, and enhanced *Mgl2* mRNA expression induced by LPS. Furthermore, HG2 significantly inhibited the increases in *Cd80*, *Cd86*, and *Nos2* mRNA expression triggered by LPS combined with IFN-γ, as well as the increases in *Cd206*, *Arg1*, *Chi3l3*, and *Mgl2* mRNA expression stimulated by IL-4. Western blotting findings supported HG2’s inhibitory effect on iNOS expression in RAW264.7 cells induced by LPS or LPS combined with IFN-γ, and the expression of CD206 and Arg-1 in RAW264.7 cells induced by IL-4. Flow cytometry (**Figures 5E–G**) and immunocytochemistry (**Supplementary Figure S5C**) corroborated the effects of HG2 on the regulation of macrophage polarization.

Interestingly, western blotting demonstrated that HG2 dose-dependently increased the expression of total COX-2 and intracellular CD80 in RAW264.7 cells. Flow cytometry revealed that HG2 dose-dependently increased MHC-II expression in RAW264.7 cells, and the effect was not influenced by LPS or LPS combined with IFN-γ or IL-4 (**Figures 6B**, **5G**).

Therefore, these findings suggested that HG2 has nuanced effects on macrophage polarization, not only influencing M1 and M2 polarization, but also promoting the transition of macrophages to a state intermediate between M1 and M2. Additionally, HG2 enhances the expression of total COX-2, intracellular CD80, and MHC-II, while inhibiting the release of inflammatory factors induced by LPS or LPS combined with IFN-γ. Thus, HG2 may modulate immune responses by affecting the antigen presentation function of macrophages.

### HG2 alleviates BLM-induced pulmonary fibrosis by improving lung function and tissue structure

**Figure 7:**
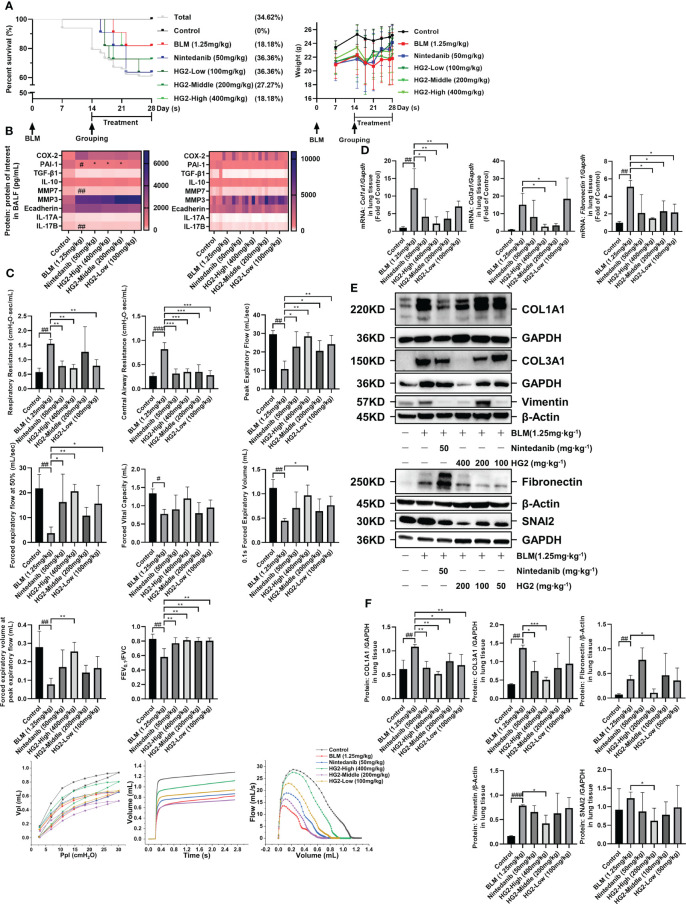
HG2 ameliorates alterations in lung function in mice with BLM-induced pulmonary fibrosis. **(A)** Survival curves and body weight changes in mice over 28 days. **(B)** Expression of factors in the BALF of mice, measured by ELISA. **(C)** After 14 days of bleomycin modeling and administration of different doses of HG2 (400, 200, 100 mg/kg), Rrs, Crs, FVC, and PEF were measured with a Flexivent mouse lung function instrument before dissection, and the pressure-volume loop and F-V loop were drawn. **(D)** Expression of ECM related mRNA in lung tissue, detected by RT-qPCR. **(E, F)** Expression of collagen I, collagen III, and laminin in mouse lung tissue, determined by western blotting. Statistical values are expressed as mean ± SD, and each experiment was independently repeated three times. Compared with the control group, ^###^p < 0.001, ^##^p < 0.01, ^#^p < 0.05; compared with the model group, ^*^p < 0.05, ^**^p < 0.01, ^***^p < 0.001.

To investigate the therapeutic potential of HG2 in pulmonary fibrosis, we used a mouse model induced by BLM and examined lung pathology and collagen deposition. Pulmonary fibrosis is characterized by restrictive ventilation disorder. Before dissection, we assessed pulmonary function with Flexivent, measurements of Rrs, Crs, PEF, FEV0.1, and FVC, and generated flow-volume (F-V) and pressure-volume curves. Compared with the control group, the model group exhibited significantly higher Rrs and Crs, and lower PEF, FVC, and FEV0.1. The F-V curve indicated restrictive and obstructive lesions, thus suggesting severe ventilation restriction in the model group. Different concentrations of HG2 and nintedanib treatment led to improved outcomes (**Figure 7C**).

We assessed the expression of factors in BALF. Compared with that in the control group, PAI-1 expression in the model group’s BALF was significantly lower. Both the nintedanib and HG2 treatment groups demonstrated clear improvement, although no significant difference was observed in the expression of other factors, possibly because of the measurement timing (28th day post-modeling) (**Figure 7B**). Furthermore, we tracked survival rates and body weight changes in the mice (**Figure 7A**).

**Figure 8:**
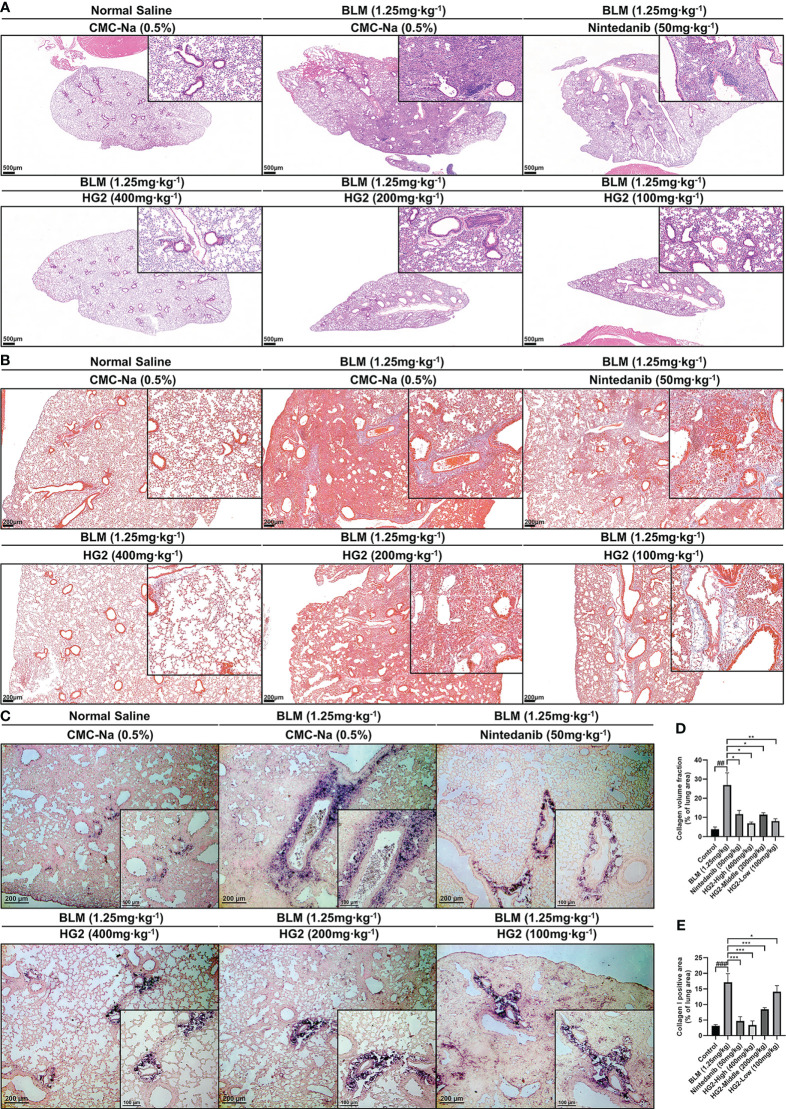
HG2 ameliorates alterations in tissue structure in mice with BLM-induced pulmonary fibrosis. **(A)** Histopathological changes in mice, visualized with HE staining. **(B)** Collagen deposition in the lungs of mice, visualized with Masson staining. **(C)** Expression of collagen I in the lungs, detected by immunohistochemistry. **(D)** Collagen volume fraction visualized with Masson staining, detected by ImageJ. **(E)** Collagen I positive area, detected by ImageJ. Scale: 500 μm **(A)**, 200 μm **(B, C)**, 100 μm **(C)**. Statistical values are expressed as mean ± SD, and each experiment was independently repeated three times. Compared with the control group, ^###^p < 0.001, ^##^p < 0.01, ^#^p < 0.05; compared with the model group, ^*^p < 0.05, ^**^p < 0.01, ^***^p < 0.001.

After 28 days of modeling, we prepared paraffin sections of lung tissue for HE staining. Pathological examination revealed alveolar and alveolar duct occlusion, interstitial thickening with fibrous tissue hyperplasia, and pulmonary bronchiolar epithelial cell exfoliation in the model group compared with the control group. Additionally, localized alveolar and alveolar duct complete occlusion, as well as interstitial fibrous tissue hyperplasia, occurred without a significant increase in dense fibers. These findings were characteristic of pulmonary fibrosis development. Lung injury was alleviated in both the HG2 and nintedanib treatment groups, and HG2 demonstrated milder effects, thus indicating superior effects against BLM-induced pulmonary fibrosis in mice (**Figure 8A**).

Massive release and deposition of ECM proteins, including collagen, laminin, and fibronectin, are characteristic of pulmonary fibrosis. Masson staining and type I collagen immunohistochemistry were used to observe collagen deposition in lung tissue sections. In contrast to the control group, the model group exhibited strong positive staining, whereas both the HG2 and nintedanib treatment groups demonstrated a notable reduction (**Figure 8B**). Immunohistochemical results also indicated specific type I collagen deposition around tracheal and vascular areas of the lungs (**Figure 8C**). ImageJ was used to semi-quantitatively detect areas in lung tissue with positive Masson and type I collagen staining. HG2 treatment at various doses, along with nintedanib treatment, showed superior anti-pulmonary fibrosis effects, and the 400 mg/kg HG2 group showed the best performance (**Figures 8D, E**).

To further validate HG2’s therapeutic effect, we assessed the expression of collagen I, collagen III, and ECM deposition-associated mRNA and proteins in the mouse lungs. The model group showed greater expression of type I collagen, type III collagen, and fibronectin mRNA than the control group. Notably, HG2 exhibited a more pronounced inhibitory effect on collagen and fibronectin mRNA expression. However, nintedanib demonstrated only anti-collagen expression effects (**Figure 7D**). These findings were corroborated by western blotting. Both HG2 and nintedanib effectively inhibited type I and type III collagen protein expression, whereas HG2 had a pronounced inhibitory effect on ECM deposition-associated proteins, such as vimentin (400 mg/kg), fibronectin (200 mg/kg), and Snail Family Transcriptional Repressor 2 (SNAI2) (200 mg/kg). In contrast, no statistically significant difference was observed in the nintedanib group (**Figures 7E, F**).

Additionally, after confirmation of HG2’s anti-BLM-induced pulmonary fibrosis effect, we conducted an acute-subacute toxicity test to preliminarily assess safety. The results revealed no acute-subacute toxicity in ICR mice (**Supplementary Tables S3–S6**).

### HG2 inhibits M2 marker expression and inflammation in macrophages in BLM-induced pulmonary fibrosis, thus modulating TGF-β/Smad pathway genes and proteins in mice

**Figure 9:**
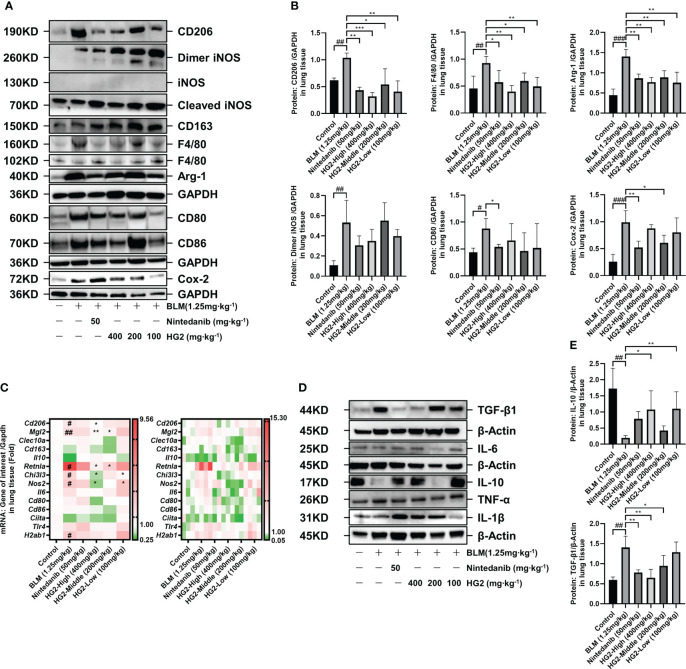
HG2 suppresses the expression of M2 markers and inflammatory factors in macrophages in the lungs of mice with BLM-induced pulmonary fibrosis, thus modulating the expression of genes and proteins in the TGF-β/Smad pathway. **(A, B)** Protein expression of M1 and M2 polarization biomarkers in mouse lung tissue. **(C)** mRNA expression of M1 and M2 polarization biomarkers in mouse lung tissue. **(D, E)** Western blot detection of the expression of inflammatory factors in mouse lung tissue. Statistical values are expressed as mean ± SD, and each experiment was independently repeated three times. Compared with the control group, ^###^p < 0.001, ^##^p < 0.01, ^#^p < 0.05; compared with the model group, ^*^p < 0.05, ^**^p < 0.01, ^***^p < 0.001.

In our investigation of the polarization state of pulmonary macrophages in mice with BLM-induced pulmonary fibrosis, we assessed the mRNA and protein expression levels of macrophage polarization-associated biomarkers. Notably, compared with the control group, the model group exhibited substantial upregulation of the M2 macrophage markers CD206 and Arg-1, as well as the macrophage marker F4/80. Intriguingly, we also observed the presence of M1 macrophage markers, including dimeric iNOS, CD80, and Cox-2. This observation was further corroborated by RT-qPCR, which revealed elevated expression levels of M2 macrophage-associated mRNA, such as *Cd206*, *Mgl2*, *Retnla*, and *Chi3l3*. Additionally, Nos2 and H2ab1 (M1 biomarkers) were also highly expressed. The presence of both M1 and M2 type macrophage markers in the model group underscores the importance of the balance between inflammation and repair and highlights the dynamic shifts in macrophage phenotype throughout idiopathic pulmonary fibrosis progression. Of note, the treatment with nintedanib and HG2 at various doses had distinct inhibitory effects on the expression of macrophage markers. The 400 mg/kg HG2 treatment demonstrated superior efficacy to nintedanib treatment (**Figures 9A–C**).

**Figure 10:**
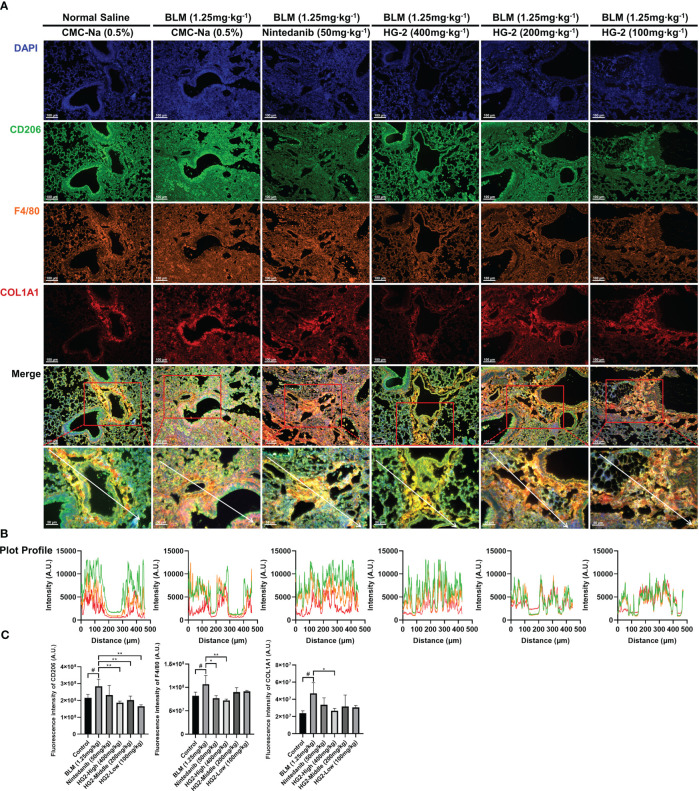
HG2 suppresses the expression of M2 markers in macrophages in the lungs of mice with BLM-induced pulmonary fibrosis. **(A)** Pathological sections of mouse left lung tissue subjected to multiple immunofluorescence staining. **(B)** Fluorescence co-localization was analyzed by Plot Profile. **(C)** Expression of collagen I, F4/80, and CD206 in mouse tissues were detected by ImageJ. Scale: 100 μm **(A)**, 50 μm **(A)**. Statistical values are expressed as mean ± SD, and each experiment was independently repeated three times. Compared with the control group, ^#^p < 0.05; compared with the model group, ^*^p < 0.05, ^**^p < 0.01.

We sought to visually depict the intricate relationship between pulmonary fibrosis and lung macrophages, particularly the M2 subtype, by using a multi-fluorescence immunohistochemical staining approach targeting CD206, F4/80, and collagen I. Our findings revealed substantial accumulation of collagen I surrounding the trachea and interstitial spaces in the model group, accompanied by macrophage infiltration and elevated CD206 expression. Encouragingly, both the HG2 treatment group and the nintedanib treatment group demonstrated significant decreases in collagen deposition, macrophage infiltration, and CD206 expression. This observation was further corroborated by fluorescence intensity analysis, which revealed markedly higher levels of CD206, F4/80, and collagen I in the model group than the control group. Notably, the 400 mg/kg HG2 group demonstrated a substantial decrease in fluorescence intensity. Additionally, qualitative analysis with Plot Profile indicated clear co-localization of collagen I, CD206, and F4/80 (**Figures 10A–C**). This comprehensive visualization provided crucial insights into the dynamic interplay among lung macrophages, collagen deposition, and the development of pulmonary fibrosis.

Given the absence of significant disparities in factors within the BALF, we sought to gain deeper insights into the effects of macrophage polarization on the expression of pulmonary inflammatory factors in mice with pulmonary fibrosis. To this end, we used western blotting to assess the levels of TGF-β, TNF-α, IL-6, IL-10, and IL-1β in lung tissue (**Figures 9D, E**). In comparison to the control group, the model group exhibited markedly higher levels of TGF-β in lung tissue, alongside significantly diminished levels of IL-10. Notably, various doses of HG2 and nintedanib demonstrated restorative effects: the 400 mg/kg and 200 mg/kg HG2 groups, as well as the nintedanib treatment group, exhibited a substantial decrease in TGF-β expression. In contrast, the 400 mg/kg and 100 mg/kg HG2 groups displayed a notable increase in IL-10 expression. These compelling results strongly indicated that HG2 modulates the expression of pulmonary inflammatory factors in mice with pulmonary fibrosis.

**Figure 11:**
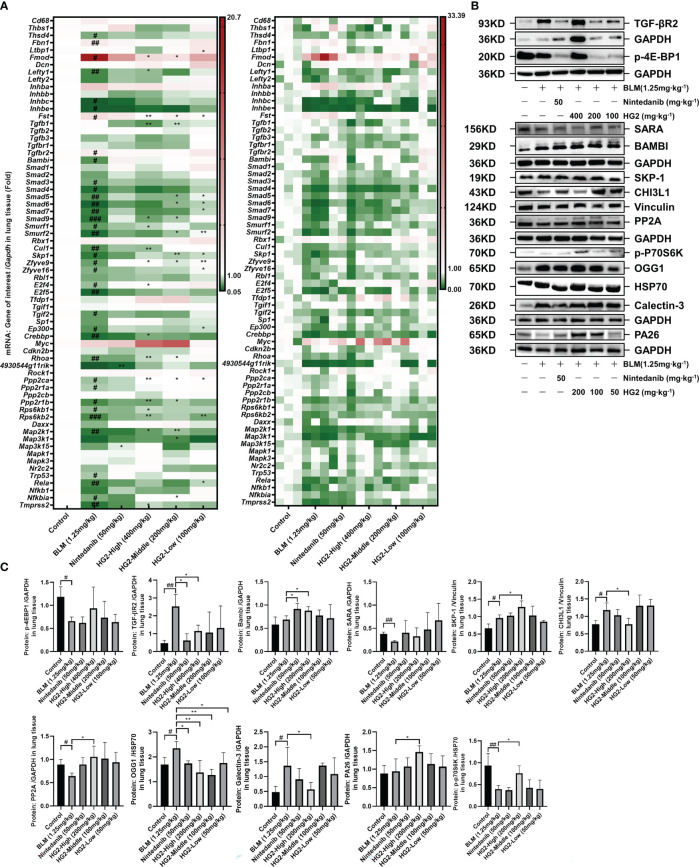
HG2 modulates the expression of genes and proteins in the TGF-β/Smad pathway. **(A)** mRNA expression of TGF-β/Smad pathway members, determined with RT-qPCR. **(B, C)** Expression of TGF-β/Smad pathway related proteins analyzed by western blotting. Statistical values are expressed as mean ± SD, and each experiment was independently repeated three times. Compared with the control group, ^###^p < 0.001, ^##^p < 0.01, ^#^p < 0.05; compared with the model group, ^*^p < 0.05, ^**^p < 0.01.

TGF-β has a critical role in both macrophage polarization and the development of pulmonary fibrosis: it is synthesized and secreted by M2 macrophages and induces further M2 polarization, thereby performing diverse functions including anti-inflammatory, pro-repair, and pro-fibrotic activities. We conducted RT-qPCR analyses to assess the TGF-β/Smad pathway gene expression levels in mice with BLM-induced pulmonary fibrosis. Compared with the control group, the BLM-induced pulmonary fibrosis group exhibited significantly higher expression of *Fmod* and *Tgfbr2* mRNA; in contrast, the mRNA expression levels of *Bambi*, *Skp1*, *Zfyve9*, *Zfyve16*, *Ppp2ca*, *Ppp2r1a*, *Ppp2r1b*, *Rps6kb1*, and *Rps6kb2* were markedly lower. Interestingly, HG2 exhibited a clear restorative effect (**Figure 11A**). *Ppp2Ca*, *Ppp2r1b*, and *Rps6kb2* demonstrated the most pronounced response.

Building on the qPCR findings, we examined protein expression profiles through western blot analysis (**Figures 11B, C**, **Supplementary Figure S5B**). In contrast to the control mice, mice with pulmonary fibrosis exhibited notably higher levels of lung proteins such as TGF-βR2, SKP-1, CHI3L1, OGG1, and Galectin-3, alongside lower expression of p-4Ebp1, SARA, p-P70S6K, and PP2A. Both nintedanib and the various doses of HG2 displayed clear restorative effects, and the high-dose HG2 group exhibited the most pronounced response.

Our results thus indicated that HG2 significantly modulates pulmonary macrophage polarization and the expression of inflammatory factors in mice with BLM-induced pulmonary fibrosis. This effect appeared to be more potent than that of nintedanib. The co-localization of CD206, F4/80, and collagen I fluorescence signals underscored the importance of assessing pulmonary macrophage polarization in pulmonary fibrosis, particularly the M2 subtype. Notably, despite the elevated expression of M2 macrophage markers in the lungs of mice with pulmonary fibrosis, the elevated levels of dimeric iNOS, CD80, and COX-2 implied that chronic pulmonary inflammation might persist in the pathogenesis of pulmonary fibrosis. HG2 and nintedanib exhibited distinct anti-inflammatory effects from those previously reported, and HG2 was found to demonstrate a more pronounced anti-inflammatory effect (**Figures 7B**, **8A**, **9A–E**). Furthermore, HG2 exerted an anti-fibrotic role by influencing the expression levels of proteins and genes in the TGF-β/Smad pathway in mice with pulmonary fibrosis. The western blotting results also prompted us to focus on apoptosis and oxidative stress in the lungs of mice with pulmonary fibrosis, where HG2 actively intervenes. Therefore, HG2, an extract from traditional Chinese medicine, targets multiple regulatory facets across various networks.

## Discussion

Macrophages play major roles in the pathogenesis of various lung diseases, extending beyond IPF and including conditions such as liver fibrosis, renal fibrosis, and skin and muscle scarring. Although the specific types of macrophages involved may differ among diseases—for example, alveolar and interstitial macrophages are prominent in pulmonary fibrosis, and Kupffer cells are prominent in liver fibrosis—their effects on fibrosis development remain remarkably similar. Moreover, the polarization state of macrophages and its influence on the immune microenvironment are intricately associated with disease progression (46–49). Throughout the fibrotic process, macrophages do not strictly favor either M1 or M2 polarization, instead, their polarization equilibrium is contingent on time. In the initial stages of injury, marked by the extensive release of inflammatory mediators and the recruitment of inflammatory monocytes, macrophages tend toward M1 polarization, corresponding to an acute inflammatory phase. Activated M1 macrophages exacerbate tissue inflammation, thereby potentially leading to significant damage. Subsequently, the expression of anti-inflammatory factors such as TGF-β increases. With the recruitment of Th2 and Treg cells, macrophages transition toward the anti-inflammatory M2 phenotype, which fosters tissue repair. However, if this reparative process becomes excessive, fibrosis may result. Our observations revealed a notable abundance of M2 macrophages in the lungs of patients with IPF, which was accompanied by an increase in the infiltration of T follicular helper cells (**Figure 1B**, **Supplementary Figure S1C**). These findings suggest an excessive reparative response involving immune cells in the lungs of patients with IPF (43, 50, 51). Further investigation is warranted to determine whether this reparative function exerts biological effects through a repair network established through cross-talk among immune cells or between immune cells and other cell types, similar to the inflammatory network in the body’s inflammatory response (52). Additionally, our bioinformatics analysis suggested that macrophage polarization in the lungs of patients with IPF represents a spectrum shift rather than a discrete two-stage transformation.

Macrophages have distinct subtypes, including M1, M2a, M2b, and M2c (41). Notably, M2a macrophages, characterized by high expression of CD206, MGL1/2, Arg-1, and Ym1, are widely acknowledged as the primary macrophage subset driving fibrosis. We observed elevated levels of CD206, MGL1/2, Arg-1, and Ym1 genes and proteins in the lung tissue in mice with BLM-induced pulmonary fibrosis, in agreement with previously reported findings (**Figures 9A–C**).

M2c macrophages promote the release of TGF-β and IL-10, and facilitate inflammation regression and tissue remodeling. Interestingly, we observed an inconsistency in the expression of TGF-β and IL-10 in the lungs of BLM-induced pulmonary fibrosis mice, wherein TGF-β was significantly upregulated and was accompanied by a notable decrease in IL-10 expression (**Figures 9D, E**), corroborating findings in the literature (53–56). Therefore, augmented polarization toward M2a may occur in macrophages, coupled with diminished M2c polarization during the progression of pulmonary fibrosis. This corresponds to the time-dependent balance of polarization described earlier, warranting further evidence for validation.

**Figure 12:**
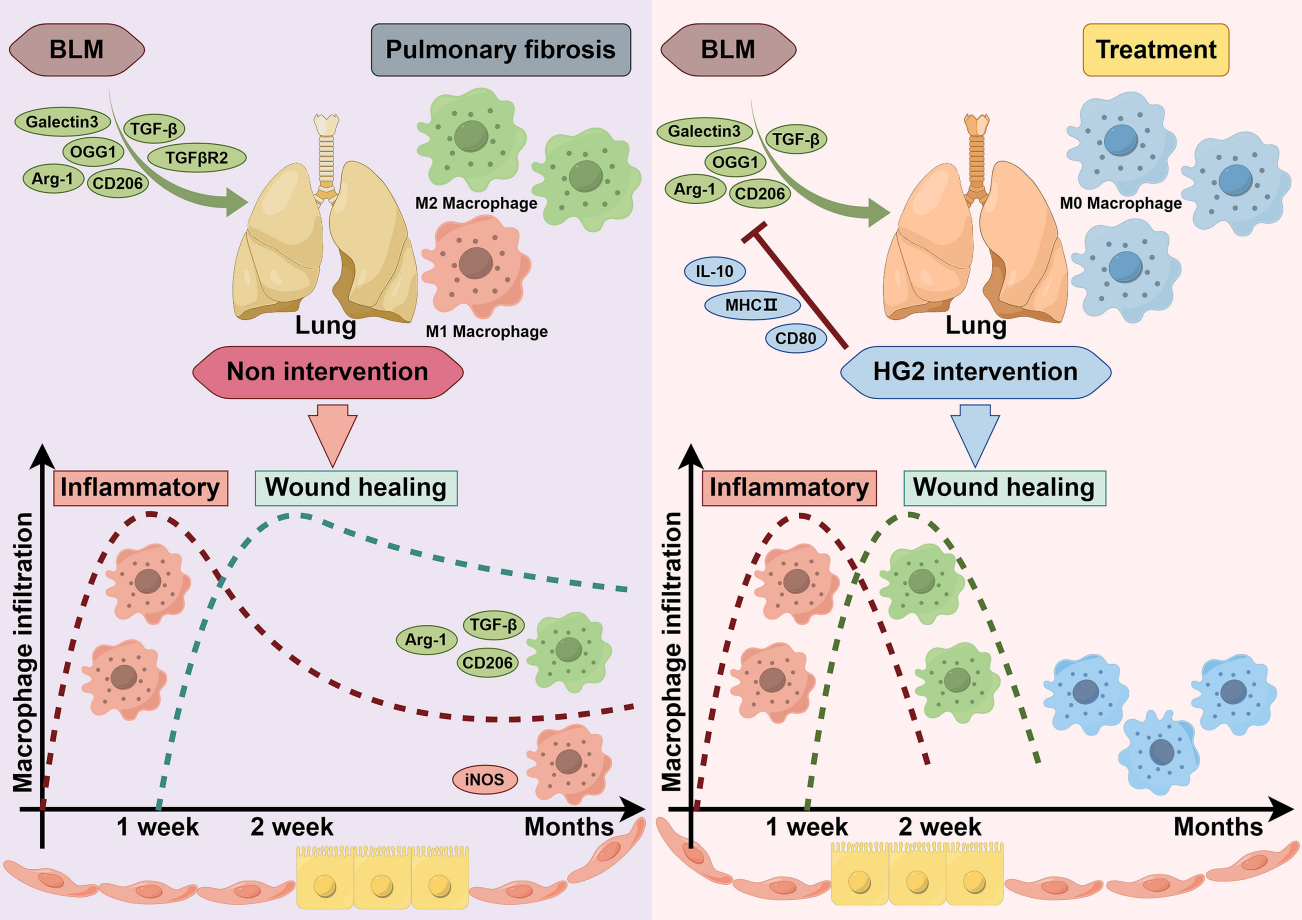
HG2 modulates the polarization balance of pulmonary macrophages and alleviates BLM-induced pulmonary fibrosis.In a healthy state, a delicate equilibrium of macrophage polarization is maintained within the lungs. However, when the lungs undergo injury induced by bleomycin, they initially exhibits an escalated inflammatory response characterized by the activation and polarization of macrophages toward the M1 phenotype. Simultaneously, the inflammatory factors released by these activated macrophages recruit a diverse array of immune cells, including macrophages, monocytes, neutrophils, eosinophils, and T cells, as well as fibroblasts and myofibroblasts. As the secretion of inflammatory cytokines intensifies, so does the release of anti-inflammatory cytokines, such as TGF-β and IL-10, thus marking the onset of lung repair. Subsequently, a shift in macrophage polarization toward the M2 phenotype occurs, thereby increasing the synthesis and deposition of extracellular matrix (ECM) components, and ultimately leading to resolution of the initial inflammation. However, in the lungs of mice with bleomycin-induced pulmonary fibrosis, aberrant expression of iNOS and Nos2 indicates incomplete resolution of the inflammatory response, which transitions from acute inflammation to chronic inflammation. This prolonged inflammation sustains the body’s repair processes, thus resulting in excessive production of TGF-β and ECM deposition, and culminating in the development of pulmonary fibrosis. HG2 regulates the polarization balance of pulmonary macrophages and facilitates pulmonary microenvironmental homeostasis through various mechanisms. On the one hand, HG2 curtails the polarization of macrophages toward the M1 phenotype, and consequently decreases the production of inflammatory cytokines and mitigates inflammatory cell infiltration. On the other hand, HG2 suppresses the M2 polarization of macrophages and consequently decreases TGF-β production. This regulation extends downstream of the TGF-β/Smad pathway, and influences the expression of genes and proteins associated with ECM deposition. HG2 consequently restricts ECM deposition, and finely modulates the onset and conclusion of pulmonary inflammatory responses and repair processes (F). Ultimately, this treatment alleviates the imbalance in inflammation-repair dynamics induced by bleomycin, thus resulting in anti-pulmonary fibrotic effects. Created with Figdraw.

HG2 robustly regulated macrophage polarization (**Figure 12**). Specifically, it not only inhibited M1 polarization induced by LPS or LPS combined with IFN-γ *in vitro*, along with M2 polarization induced by IL-4, but also restrained the expression of CD206, MGL1/2, Arg-1, and Ym1 gene transcription and protein translation in mice with BLM-induced pulmonary fibrosis *in vivo* (**Figures 5**, **6**, **9A–C**). Additionally, HG2 exerted an unexpected dose-dependent increase in MHC-II and CD80 *in vitro*, which was not affected by the various stimulation conditions (**Figures 5F**, **6B**). This may potentially enhance the antigen presentation capability of macrophages and bolster cellular immunity, thereby reflecting the intricate regulatory effects of HG2 on macrophages.

One limitation of our study is that we did not specifically investigate whether HG2 treatment mitigates pulmonary inflammation and fibrosis 1 week after BLM model establishment. However, HG2 demonstrated pronounced anti-inflammatory effects *in vitro*, particularly in relation to the secretion of IL-6, IL-10, TNF-α, and TGF-β, as well as the release of NO and the expression of iNOS (**Figures 5**, **6**). Therefore, HG2 might have applications not only in fibrosis treatment but also in addressing various lung diseases, particularly inflammatory conditions such as asthma and acute exacerbation of chronic obstructive pulmonary disease. This key direction will be a focus of our future research.

Furthermore, our findings highlighted aberrant expression of dimeric iNOS, CD80, and COX-2 proteins, along with the Nos2 gene in the lung tissue of mice with BLM-induced pulmonary fibrosis. However, no discernible differences were noted in the expression of IL-6, IL-1β, or TNF-α, which are commonly used indicators of acute inflammation (**Figures 9A, D**). Therefore, although acute inflammation may subside in the pulmonary fibrosis stage, chronic inflammation may persist and potentially contribute to the sustained expression of TGF-β. In addition, a dichotomous view of macrophage polarization has influenced understanding of the role of macrophages in pulmonary fibrosis (57–61). Interventions targeting chronic inflammation may be a viable approach for addressing pulmonary fibrosis, in agreement with clinical observations that anti-inflammatory therapies have not significantly improved IPF outcomes. Therefore, clinical attention must be paid to chronic rather than acute inflammation in the course of IPF.

Nintedanib, an intracellular tyrosine kinase inhibitor, exerts its effects by competitively binding PDGFR-α/β, FGFR1–3, and VEGFR1–3, and effectively impeding intracellular signal transduction. Its action includes the inhibition of lymphocyte-specific tyrosine kinase (Lck), thus decreasing the release of key cytokines including IL-2, 4, 5, 10, 12p70, 13, and IFN-γ from monocytes and T cells. Additionally, nintedanib suppresses the secretion of CCL2 and 18. Notably, nintedanib exerts inhibitory effects on fibroblasts, by hampering their migration, differentiation, and proliferation, and also hinders the transformation of fibroblasts into myofibroblasts, along with the release of MMPs and collagen induced by TGF-β. Furthermore, nintedanib impedes the proliferation of vascular endothelial cells, pericytes, and smooth muscle cells, and it exhibits favorable effects on the interaction between pulmonary mast cells and fibroblasts in people with IPF (62–64).

Proteomic findings underscored the distinctions between HG2 and nintedanib. In comparison to nintedanib, HG2 was found to target primarily immune regulation and collagen inhibition (**Supplementary Figure S4A**). Our observations confirmed nintedanib’s ability to inhibit pulmonary macrophage infiltration in mice with BLM-induced pulmonary fibrosis. This treatment potently inhibited the M2 macrophage polarization biomarkers CD206 and Arg-1, while promoting the release of IL-10. This finding aligns with the previously documented *in vitro* inhibitory effect of nintedanib on M2 macrophage polarization (65). Unlike HG2, nintedanib did not significantly inhibit *Nos2* gene expression.

Traditional Chinese medicine takes a holistic approach, emphasizing that “the lung is a delicate organ, and imbalances in lung heat or cold can lead to weakness.” In the context of IPF, deficiencies in Qi, yin, and yang in the lungs are believed to exist and to be further influenced by factors such as phlegm, heat, stasis, and toxins (66, 67). Treatment for IPF focuses on a dialectical approach, with a multi-target, multi-level, low toxicity nature, to address symptoms, affect molecular expression, and regulate microenvironmental balance (68, 69).

We posit that HG2’s multi-target and wide-ranging effects, including its anti-inflammatory properties and regulatory effects on polarization, align with the traditional Chinese medicine principle of addressing both symptoms and underlying causes, and with the concept of the “drug-cloud” (70). Although another limitation of our study is that we did not investigate the effects of HG2 on the well-recognized JAK/STAT signaling pathway, which regulates macrophage polarization, HG2 nonetheless demonstrated a notable regulatory effect on macrophage polarization. This effect is crucial for maintaining the immune microenvironment balance in the lungs and supporting the re-establishment of immune equilibrium in IPF progression.

HG2’s effects on the TGF-β/Smad pathway and downstream proteins highlighted its effects on established molecular events influencing IPF (**Figure 12**). Additionally, its effects in improving lung function, decreasing collagen deposition, and inhibiting inflammatory factors released by macrophages underscore its symptom-alleviating effects. This multifaceted, multi-level role sets HG2 apart from nintedanib. Furthermore, the results of acute-subacute toxicity tests confirmed HG2’s high safety profile. Because of its low toxicity, multi-target approach, homeostatic effects, and cost-effectiveness, we believe that HG2 warrants further investigation and ongoing development, particularly in comparison to nintedanib.

In summary, our study identified a promising new extract, HG2, derived from *Prismatomeris connata* Y. Z. Ruan, which demonstrated significant anti-BLM-induced pulmonary fibrosis effects. Through a series of *in vitro* and *in vivo* experiments, we established that HG2 modulates macrophage polarization and exerts anti-inflammatory properties. Our examination of the role of macrophages in the pathogenesis of IPF revealed HG2’s notable ability to inhibit M2 macrophage polarization during BLM-induced pulmonary fibrosis and effectively suppress LPS or LPS combined with IFN-γ-induced macrophage inflammation and M1-type polarization. Furthermore, our investigation of HG2’s effects on the downstream TGF-β/Smad pathway, a critical component in the macrophage polarization cascade, and on subsequent downstream targets, highlighted HG2’s multifaceted and wide-ranging functionality. Our findings suggest that HG2 has substantial promise as a potential next-generation anti-pulmonary fibrosis drug, which warrants further in-depth research and development.

## Data availability statement

The original contributions presented in the study are included in the article/**Supplementary Material**. The data presented in the study are deposited in the ProteomeXchange repository, accession number PXD048007. Further inquiries can be directed to the corresponding authors.

## Ethics statement

Ethical approval was not required for the study involving humans in accordance with the local legislation and institutional requirements. Written informed consent to participate in this study was not required from the participants or the participants’ legal guardians/next of kin in accordance with the national legislation and the institutional requirements. The animal study was approved by Experimental Animal Welfare Ethics Committee of Beijing Union-Genius Pharmaceutical Technology Development Co., Ltd. The study was conducted in accordance with the local legislation and institutional requirements.

## Author contributions

SL: Writing – original draft, Writing – review & editing, Formal analysis, Methodology, Validation, Visualization. GH: Writing – review & editing, Writing – original draft, Formal analysis, Methodology, Visualization. LK: Writing – review & editing, Methodology. TZ: Writing – review & editing, Methodology. HYJ: Writing – review & editing, Methodology. FP: Writing – review & editing, Methodology. JL: Writing – review & editing, Methodology. XC: Writing – review & editing, Methodology. JB: Writing – review & editing, Methodology. WL: Writing – review & editing, Methodology. CL: Writing – review & editing, Methodology. ML: Writing – review & editing, Methodology. LW: Writing – review & editing, Methodology. DZ: Writing – review & editing, Methodology. JZ: Writing – review & editing, Methodology. ZY: Writing – original draft, Writing – review & editing, Data curation, Formal analysis, Methodology, Project administration. HTJ: Writing – original draft, Writing – review & editing, Data curation, Formal analysis, Funding acquisition, Methodology, Project administration, Supervision.
