# Supplementary material for: Unraveling the mechanism of ethyl acetate extract from Prismatomeris connata Y. Z. Ruan root in treating pulmonary fibrosis: insights from bioinformatics, network pharmacology, and experimental validation
Source: Front Immunol. 2024 Jan 8;14:1330055. doi: 10.3389/fimmu.2023.1330055 (PMC10801734; doi:10.3389/fimmu.2023.1330055)
Supplement: Supplementary file 1 [file DataSheet_1.docx]

# Title: Unraveling the mechanism of ethyl acetate extract from *Prismatomeris connata* Y. Z. Ruan root in treating pulmonary fibrosis: insights from bioinformatics, network pharmacology, and experimental validation

**Supplementary data**

**Table S1 Brands of primary antibodies and secondary antibodies.**

| **Experiment** | **Name** | **Brand/Country** | **Cat.** |
| --- | --- | --- | --- |
|  | Collagen Ⅰ | Abcam/England | ab279711 |
|  | Collagen Ⅲ | Boster/China | A00788-3 |
|  | Vimentin | Proteintech/China | 10366-1-AP |
|  | Fibronectin | Proteintech/China | 15613-1-AP |
|  | SNAI2 | Proteintech/China | 12129-1-AP |
|  | GAPDH | Cell Signaling Technology/USA | 5174S |
|  | β-Actin | Cell Signaling Technology/USA | 4970S |
|  | CD206 | Cell Signaling Technology/USA | 24595S |
|  | iNOS | Cell Signaling Technology/USA | 2982S |
|  | CD163 | Boster/China | A00812-2 |
|  | F4/80 | Proteintech/China | 28463-1-AP |
|  | Arg-1 | Cell Signaling Technology/USA | 93668T |
|  | CD80 | Proteintech/China | 66406-1-Ig |
|  | CD86 | Proteintech/China | 13395-1-AP |
|  | Cox-2 | Cell Signaling Technology/USA | 12282S |
|  | TGF-β1 | Cell Signaling Technology/USA | 3709S |
|  | IL-10 | Boster/China | RP1015 |
|  | IL-6 | Abcam/England | ab229381 |
|  | TNF-α | Abcam/England | ab183218 |
|  | IL-1β | Abcam/England | ab200478 |
|  | TGF-βR2 | Cell Signaling Technology/USA | 79424T |
|  | p-4E-BP1 | Cell Signaling Technology/USA | 2855T |
|  | SARA | Proteintech/China | 22033-1-AP |
|  | BAMBI/NMR | Abcam/England | ab200737 |
| **Western Blot** | SKP-1 | Proteintech/China | 10990-2-AP |
|  | CHI3L1 | Abcam/England | ab259322 |
|  | Vinculin | Boster/China | A01207-1 |
|  | PPP2Ca | Proteintech/China | 13482-1-AP |
|  | p-P70S6K (Thr389) | Cell Signaling Technology/USA | 9234T |
|  | OGG1 | ImmunoWay/USA | YN3038 |
|  | HSP70 | Proteintech/China | 25405-1-AP |
|  | Galectin-3 | Sigma-Aldrich/Germany | 310203 |
|  | PA26 | ImmunoWay/USA | YT3562 |
|  | Samd4 | Proteintech/China | 10231-1-AP |
|  | p-mTOR | Cell Signaling Technology/USA | 5536T |
|  | mTOR | Cell Signaling Technology/USA | 2983T |
|  | Cul-1 | Proteintech/China | 12895-1-AP |
|  | TMPRSS2 | Proteintech/China | 14437-1-AP |
|  | HIF-1α | Abcam/England | ab216842 |
|  | Ecadherin | ImmunoWay/USA | YT1454 |
|  | VEGFR2 | Abcam/England | ab221679 |
|  | GDF-8 | Abcam/England | ab124721 |

|  | PAI-1 | Cell Signaling Technology/USA | 27535S |
| --- | --- | --- | --- |
|  | SFTPC | Abcam/England | ab211326 |
|  | PGC-1α | Boster/China | BM4898 |
|  | CD68 | Abcam/England | ab955 |
|  | PDRG1 | ImmunoWay/USA | YT3648 |
|  | Anti-rabbit IgG, HRP-linked | Cell Signaling Technology/USA | 7074 |
|  | Antibody |  |  |
| **Multiple fluorescence** | CD206 | Cell Signaling Technology/USA | 24595S |
| **immunohistochemistry** | F4/80 | Proteintech/China | 28463-1-AP |
|  | Collagen Ⅰ | Abcam/England | ab279711 |
|  | CD206 | Cell Signaling Technology/USA | 24595S |
|  | CD86 | Proteintech/China | 13395-1-AP |
|  | Arg-1 | Cell Signaling Technology/USA | 93668T |
|  | iNOS | Cell Signaling Technology/USA | 2982S |
| **Immunocytochemistry** | CD163  CD80 | Boster/China  Proteintech/China | A00812-2  66406-1-Ig |
|  | Goat Anti-Rabbit IgG H&L | Abcam/England | ab150077 |
|  | (Alexa Fluor® 488) |  |  |
|  | Goat Anti-Rabbit IgG H&L | Abcam/England | ab150115 |
|  | (Alexa Fluor® 647) |  |  |

**Table S2 Primer sequence of RT-qPCR detection gene.**

| **Name** | **Forward Sequence (5’-3’)** | **Reverse Sequence (5’-3’)** |
| --- | --- | --- |
| Mouse *Gapdh* | CCATCACCATCTTCCAGG | AGACTCCACGACATACTCA |
| Mouse *Col1a1* | GCTCCTCTTAGGGGCCACT | CCACGTCTCACCATTGGGG |
| Mouse *Col3a1* | ACGTAGATGAATTGGGATGCAG | GGGTTGGGGCAGTCTAGTG |
| Mouse *Fibronectin* | GCTCAGCAAATCGTGCAGC | CTAGGTAGGTCCGTTCCCACT |
| Mouse *Cd206* | CTCTGTTCAGCTATTGGACGC | TGGCACTCCCAAACATAATTTGA |
| Mouse *Mgl2* | TTAGCCAATGTGCTTAGCTGG | GGCCTCCAATTCTTGAAACCT |
| Mouse *Clec10a* | TGAGAAAGGCTTTAAGAACTGGG | GACCACCTGTAGTGATGTGGG |
| Mouse *Cd163* | ATGGGTGGACACAGAATGGTT | CAGGAGCGTTAGTGACAGCAG |
| Mouse *Il10* | TTAATAAGCTCCAAGACCAAGG | CATCATGTATGCTTCTATGCAG |
| Mouse *Retnla* | CCAATCCAGCTAACTATCCCTCC | CCAGTCAACGAGTAAGCACAG |
| Mouse *Chi3l3* | CAGGTCTGGCAATTCTTCTGAA | GTCTTGCTCATGTGTGTAAGTGA |
| Mouse *Nos2* | GTTCTCAGCCCAACAATACAAGA | GTGGACGGGTCGATGTCAC |
| Mouse *Il6* | TCTCTGCAAGAGACTTCCA | CGGACTTGTGAAGTAGGGA |
| Mouse *Cd80* | ACCCCCAACATAACTGAGTCT | TTCCAACCAAGAGAAGCGAGG |
| Mouse *Cd86* | TCAATGGGACTGCATATCTGCC | GCCAAAATACTACCAGCTCACT |
| Mouse *Ciita* | TGCGTGTGATGGATGTCCAG | CCAAAGGGGATAGTGGGTGTC |
| Mouse *Tlr4* | ATGGCATGGCTTACACCACC | GAGGCCAATTTTGTCTCCACA |
| Mouse *H2ab1* | AGCCCCATCACTGTGGAGT | GATGCCGCTCAACATCTTGC |
| Mouse *Tgfb1* | CTCCCGTGGCTTCTAGTGC | GCCTTAGTTTGGACAGGATCTG |
| Mouse *Arg1* | CTCCAAGCCAAAGTCCTTAGAG | AGGAGCTGTCATTAGGGACATC |
| Mouse *Cd68* | CTTTGGATTCAAACAGGACCT | AAGGACACATTGTATTCCACC |
| Mouse *Thbs1* | TGAGAATGCCAACCTGAT | GGAGACCACACTGAAGAT |
| Mouse *Thsd4* | GTGACTCCAGCATGAAGCC | TCTTGCTGCACTCCGACCA |
| Mouse *Fbn1* | TGAAGATGTGGACGAGTGT | TTGGTAGTGCTGGAGGTAG |
| Mouse *Ltbp1* | CCGATGAGAACCAGGAGTA | GCCAGGACATTATCACAGAG |
| Mouse *Fmod* | GTCTCACTAACAACGGCCT | TTGACAGGAGGGATCTTCT |
| Mouse *Dcn* | CTACCTTCACAACAACAACATC | AGAACGCACATAGACACATC |
| Mouse *Lefty1* | CTGGACAAGGCTGATGTGG | TGGCATGGCTGTGTTGTAG |
| Mouse *Lefty2* | CACAAGTTGGTCCGTTTCG | CTCCGTAGTCCTTGAGGTC |
| Mouse *Inhba* | CTCTCCTTCCACTCAACA | ACACTCCTCCACAATCAT |
| Mouse *Inhbb* | CTTCGTCTCTAATGAAGGCAACC | CTCCACCACATTCCACCTGTC |
| Mouse *Inhbc* | CTCCAACCACAGTAGTGAACC | CACTGGCCGACTGAGTATGG |
| Mouse *Inhbe* | CAGAATAACTCGGCCTCTG | TGGATTTGTCTATGATGGTAGC |
| Mouse *Fst* | CAGACCAATAATGCCTACT | AGTAAGTCACTCCATCATT |
| Mouse *Tgfb2* | TCGACATGGATCAGTTTATGCG | CCCTGGTACTGTTGTAGATGGA |
| Mouse *Tgfb3* | CCTGGCCCTGCTGAACTTG | TTGATGTGGCCGAAGTCCAAC |
| Mouse *Tgfbr1* | CCTTGAGAGTGATGGCTAA | CTTCCTGTTGGCTGAGTT |
| Mouse *Tgfbr2* | ATCGCTCATCTCCACAGT | CAGGCAACAGGTCAAGTC |
| Mouse *Bambi* | AGCATGACAGCAGCAGAAA | AACATAATGAGCAGCACCA |
| Mouse *Smad1* | ACTGGTGCTCTATTGTGTA | GCGGTTCTTATTGTTGGA |
| Mouse *Smad2* | TCCGTACCACTACCAGAGAGT | GGCGGCAGTTCTGTTAGAATC |
| Mouse *Smad3* | AGGGGCTCCCTCACGTTATC | CATGGCCCGTAATTCATGGTG |

| Mouse *Smad4* | CCTGTTGTGACTGTGGAT | TGGACATTGGAGAGTTGAC |
| --- | --- | --- |
| Mouse *Smad5* | TTGTTCAGAGTAGGAACTGCAAC | GAAGCTGAGCAAACTCCTGAT |
| Mouse *Smad6* | TACAAGCCACTGGATCTGT | TGAGAATTCACCCGGAGCA |
| Mouse *Smad7* | GGCCGGATCTCAGGCATTC | TTGGGTATCTGGAGTAAGGAGG |
| Mouse *Smad9* | CGGGTCAGCCTAGCAAGTG | GAGCCGAACGGGAACTCAC |
| Mouse *Smurf1* | AGCATCAAGATCCGTCTGACA | CCAGAGCCGTCCACAACAAT |
| Mouse *Smurf2* | CTTGGTTGTGTTCGTCTTCT | CGTGCCTATTCGGTCTCT |
| Mouse *Rbx1* | TTGATAACTGTGCCATCTG | GACTCCCCATGCAACCGTA |
| Mouse *Cul1* | GATGAGAGTGTCCTGAAGTT | GGTAGGCACAGATTCCATT |
| Mouse *Skp1* | TGCTGGAAGATTTGGGAAT | CACCACTGAATGACCTTCT |
| Mouse *Zfyve9* | CCAGTCCTCTTCCAACAG | TTCCACAGCATAATCTCCTT |
| Mouse *Zfyve16* | AAGTGAGATAAGCCAGAGT | GGACAACAGTAGGAGTAGA |
| Mouse *Rbl1* | GCCAATGTCTCCAATAATAC | GTCTCCTCTTAGCACTTC |
| Mouse *E2f4* | CCTGGAACTGAGAACAAG | GCTGCTGCTACTACTATC |
| Mouse *E2f5* | CCACCAAATTCGTGTCGTTGC | AAATTCTTCGCTTTTGCCTCAC |
| Mouse *Tfdp1* | TAGAACCATACGTGACAGA | TCAAATCACTGGCAGAAAG |
| Mouse *Tgif1* | GCAAGAGAAGGAGGAGAG | TAGGCGTTGTATCTGTGTT |
| Mouse *Tgif2* | TCGACATGGATCAGTTTATGCG | CCCTGGTACTGTTGTAGATGGA |
| Mouse *Sp1* | CCAATGAGAACAGCAACA | CCAGAGGAGGAAGAGATG |
| Mouse *Ep300* | TTCAGCCAAGCGGCCTAAA | CGCCACCATTGGTTAGTCCC |
| Mouse *Crebbp* | CCTGAACCTACTGAATCCA | CTCTGTCGTATCTGTCTCTT |
| Mouse *Myc* | CTCTGCTCTCCATCCTAT | GTAACTCGGTCATCATCTC |
| Mouse *Cdkn2b* | GCCCAATCCAGGTCATGAT | AGTTGGGTTCTGCTCCGTG |
| Mouse *Rhoa* | CCAGTTCCCAGAGGTCTAT | GGCGGTCATAATCTTCCTG |
| Mouse *4930544g11rik* | CTGGTTGGGAGTAAGAAGGA | TCTTGGCTAACTCTTGTATCGT |
| Mouse *Rock1* | TTGCTGGATGGATTGGATG | ACCTCTGCCGATTACCTT |
| Mouse *Ppp2Ca* | TGGAGGGATATAACTGGTG | GTCGTCAAGTTCCATGATT |
| Mouse *Ppp2r1a* | GACGGTGACGATTCGCTCTAT | CTGGTCCGTTCAACCCCAAG |
| Mouse *Ppp2cb* | GAGGCTACTACTCTGTGGAGAC | CAGGGCTCTTATGTGGTCCAG |
| Mouse *Ppp2r1b* | TCGCGGTTTTAATCGACGAG | CTACCCCGAGTGCTAGAGCTA |
| Mouse *Rps6kb1* | AGACACAGCGTGCTTTTACTT | GTGTGCGTGACTGTTCCATCA |
| Mouse *Rps6kb2* | ACATCTTGAGCGAGAAGGC | TGAGGTCCCGGTAGATGAT |
| Mouse *Daxx* | ATGGAGGGAGCAGTAACTC | TCTTACACAGTTCAAGGAACTC |
| Mouse *Map2k1* | TGTGCAGTCGGACATCTGG | GCATCCAAACAGTAGCTCC |
| Mouse *Map3k1* | CTGTGATGTATGGAGTGTT | CGAGATGATTGGAGTGTT |
| Mouse *Map3k15* | GCAGTCACCATTCTCATTC | TTCTACTTCTGTGTCCTTGT |
| Mouse *Mapk1* | ACAGGGTTCTTGACAGAGT | CAGCCCACAGACCAAATAT |
| Mouse *Mapk3* | TCCGCCATGAGAATGTTATAGGC | GGTGGTGTTGATAAGCAGATTGG |
| Mouse *Nr2c2* | ATTCTTGTCCGCCTACCA | TCTGCTGTCTCCATCTTGA |
| Mouse *Trp53* | GCGTAAACGCTTCGAGATGTT | TTTTTATGGCGGGAAGTAGACTG |
| Mouse *Rela* | AGGCTTCTGGGCCTTATGTG | TGCTTCTCTCGCCAGGAATAC |
| Mouse *Nfkb1* | ATGGCAGACGATGATCCCTAC | TGTTGACAGTGGTATTTCTGGTG |
| Mouse *Nfkbia* | TGAAGGACGAGGAGTACGAGC | TTCGTGGATGATTGCCAAGTG |
| Mouse *Tmprss2* | CAGTCTGAGCACATCTGTCCT | CTCGGAGCATACTGAGGCA |

**Table S3 Blood biochemistry of ICR mice administrated with 5000mg/kg HG2 for 14 days.**

| **Item/Group** | **Control** | **Administration** |
| --- | --- | --- |
| ALT (u/L) | 51.33±17.61 | 41.56±8.58 |
| AST (u/L) | 165.00±71.13 | 126.89±29.27 |
| TP (g/L) | 60.93±4.88 | 58.67±2.02 |
| ALB (g/L) | 23.67±1.26 | 23.62±1.07 |
| GLB (g/L) | 37.27±3.76 | 35.04±1.55 |
| A/G (%) | 0.58±0.04 | 0.63±0.05 |
| TBIL (μmol/L) | 0.53±2.12 | 1.86±1.13 |
| ALP (u/L) | 70.33±9.62 | 129.56±31.48## |
| LDH (u/L) | 1270.00±310.03 | 969.33±177.78## |
| CK (u/L) | 2558.33±1748.07 | 1553.11±633.85 |
| GLU (mmol/L) | 8.57±5.62 | 7.66±0.78 |
| UREA (mmol/L) | 6.29±0.47 | 5.45±0.79# |
| UA (mmol/L) | 129.10±80.45 | 88.58±28.35 |
| CREA (μmol/L) | 9.23±1.23 | 7.98±1.86 |
| CHOL (mmol/L) | 3.16±0.57 | 3.43±0.60 |
| TG (mmol/L) | 1.30±0.43 | 2.17±0.66# |
| K (mmol/L) | 7.40±2.09 | 7.07±0.52 |
| Na (mmol/L) | 151.00±3.00 | 149.33±1.63 |
| Cl (mmol/L) | 110.67±3.77 | 109.11±2.85 |
| Ca (mmol/L) | 2.38±0.15 | 2.36±0.04 |

Sixteen SPF grade ICR female mice were chosen, with 6 assigned to the control group and 10 to the administration group. Initially, HG2 was administered intragastrically at doses of 1000mg/kg (2 mice), 2000mg/kg (4 mice), and 5000mg/kg (4 mice) for a duration of 7 days. As no fatalities or toxic reactions were observed in the animals, the experiment was modified to a subacute toxicity test, wherein the maximum dose of 5000mg/kg was administered for a period of 14 days. Blood biochemical analyses were conducted after the completion of the 14-day period. The blood biochemical analysis revealed notable disparities in ALP, LDH, UREA, and TG levels between the administration group and the control group. Conversely, no significant alterations were observed in the remaining parameters. Statistical values were expressed as mean ±SD, compared with the control group, ##p<0.01, #p<0.05, compared with the control group

**Table S4 Classification of blood cells in ICR mice administrated with 5000mg/kg HG2 for 14 days.**

| **Item/Group** | **Control** | **Administration** |
| --- | --- | --- |
| WBC (109/L) | 4.60±1.92 | 3.88±1.40 |
| RBC (109/L) | 9.10±0.65 | 8.21±0.68# |
| HGB (g/L) | 154.00±16.75 | 146.89±7.14 |
| HCT (%) | 43.20±3.65 | 37.82±3.16# |
| MCV (fL) | 54.70±14.49 | 46.03±0.88 |
| MCH (pg) | 19.40±4.89 | 17.96±1.02 |
| MCHC (g/L) | 413.83±125.84 | 390.22±26.11 |
| RDW (%) | 17.17±5.85 | 15.07±0.64 |
| PLT(109/L) | 802.33±248.75 | 883.00±120.75 |
| PCT (%) | 0.54±0.18 | 0.61±0.09 |
| MPV (fL) | 7.65±1.96 | 6.98±0.61 |
| PDW (%) | 17.35±5.60 | 14.64±0.82 |
| LYM(109/L) | 3.72±1.71 | 2.63±1.12 |
| MON(109/L) | 0.03±0.05 | 0.04±0.07 |
| NEUT(109/L) | 0.85±0.31 | 1.18±0.68 |
| EOS(109/L) | 0.00±0.00 | 0.02±0.04 |
| BAS(109/L) | 0.00±0.00 | 0.00±0.00 |
| LYM% (%) | 92.85±35.80 | 68.36±14.37 |
| MON% (%) | 0.70±0.52 | 1.46±1.09 |
| NEUT% (%) | 22.42±7.41 | 29.76±13.76 |
| EOS% (%) | 0.70±0.81 | 0.41±0.67 |
| BAS% (%) | 0.00±0.00 | 0.02±0.06 |

Sixteen SPF grade ICR female mice were chosen, with 6 assigned to the control group and 10 to the administration group. Initially, HG2 was administered intragastrically at doses of 1000mg/kg (2 mice), 2000mg/kg (4 mice), and 5000mg/kg (4 mice) for a duration of 7 days. As no fatalities or toxic reactions were observed in the animals, the experiment was modified to a subacute toxicity test, wherein the maximum dose of 5000mg/kg was administered for a period of 14 days. Blood biochemical analyses were conducted after the completion of the 14-day period. The results showed that there were no significant changes in the other items except for the significant differences in RBC and HCT. Statistical values were expressed as mean ±SD, compared with the control group, #p<0.05, compared with the control group.

**Table S5 Organ coefficient of ICR mice administrated with 5000mg/kg HG2 for 14 days.**

| **Item/Group** | **Control** | **Administration** |
| --- | --- | --- |
| Heart/Weight（%） | 0.56±0.04 | 0.61±0.07 |
| Liver/Weight（%） | 5.30±0.34 | 5.97±0.38## |
| Spleen/Weight（%） | 0.48±0.07 | 0.51±0.12 |
| Lung/Weight（%） | 0.65±0.05 | 0.84±0.16# |
| Kidney/Weight（%） | 1.24±0.03 | 1.39±0.16 |
| Left kidney/Weight（%） | 0.61±0.01 | 0.71±0.08# |
| Right kidney/Weight（%） | 0.63±0.03 | 0.68±0.09 |

Sixteen SPF grade ICR female mice were chosen, with 6 assigned to the control group and 10 to the administration group. Initially, HG2 was administered intragastrically at doses of 1000mg/kg (2 mice), 2000mg/kg (4 mice), and 5000mg/kg (4 mice) for a duration of 7 days. As no fatalities or toxic reactions were observed in the animals, the experiment was modified to a subacute toxicity test, wherein the maximum dose of 5000mg/kg was administered for a period of 14 days. Blood biochemical analyses were conducted after the completion of the 14-day period. The findings indicated a significant increase in the weight of the liver, lung, and left kidney within the administration group, while no notable changes were observed in the remaining organs. Statistical values were expressed as mean ±SD, compared with the control group, ##p<0.01, #p<0.05, compared with the control group1.

**Table S6 Pathological examination of ICR mice administrated with 5000mg/kg HG2 for 14 days.**

**Organ**

**Lesion and grading**

**Control Administration**

| **or part** |  | **♀** | **♂** | **♀** | **♂** |
| --- | --- | --- | --- | --- | --- |
| Chronic | ++ | 1/6 | / | 0/9 | / |

Lung

bronchial

Total 1/6 0/9

| inflammation |  | | | | | | |
| --- | --- | --- | --- | --- | --- | --- | --- |
| Cell  hypertrophy | ++  Total | 1/6 | 1/6 | / | 0/9 | 0/9 | / |
| Chronic inflammation | ++  Total | 0/6 | 0/6 | / | 1/9 | 1/9 | / |

Liver

Kidney

Sixteen SPF grade ICR female mice were chosen, with 6 assigned to the control group and 10 to the administration group. Initially, HG2 was administered intragastrically at doses of 1000mg/kg (2 mice), 2000mg/kg (4 mice), and 5000mg/kg (4 mice) for a duration of 7 days. As no fatalities or toxic reactions were observed in the animals, the experiment was modified to a subacute toxicity test, wherein the maximum dose of 5000mg/kg was administered for a period of 14 days. Blood biochemical analyses were conducted after the completion of the 14-day period. The findings indicated the presence of mild inflammation in the lung and bronchi, as well as mild hepatocyte hypertrophy in one animal from the control group. Additionally, mild chronic inflammation was observed in the kidney of one animal from the administration group, while no noteworthy pathological alterations were detected in the organs of the remaining animals. +: slight; ++: mild; +++: medium; ++++: heavy.


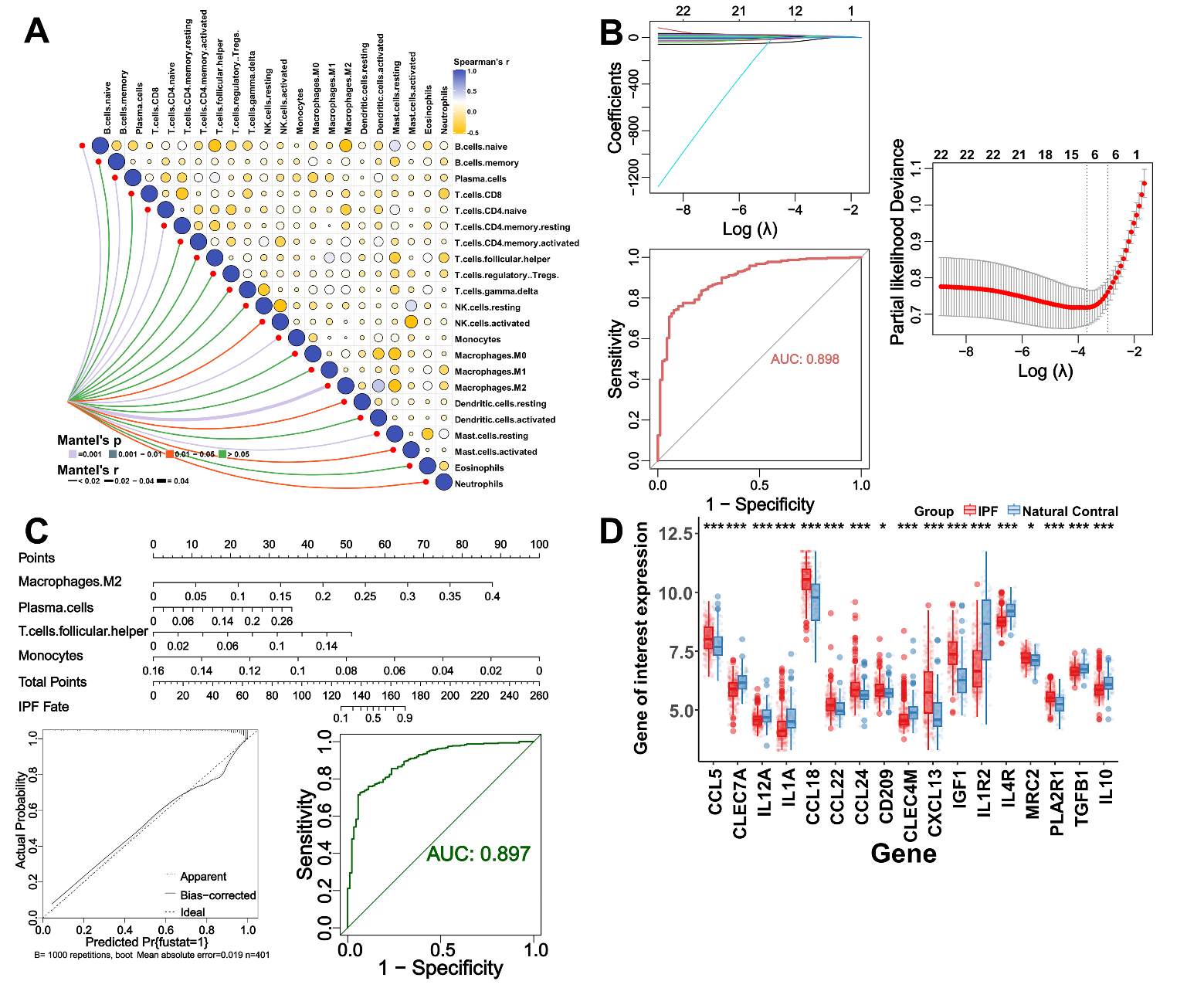


**Figure S1. Bioinformatics analysis highlights the vital crucial role of macrophage polarization in idiopathic pulmonary fibrosis.**

(A) Correlation heatmap of 22 types of immune cells in IPF lung tissue and control tissue. The correlations were determined with Spearman correlation, and the correlation of immune cells and group was determined with the Mantel test. Blue indicates a positive correlation, whereas yellow indicates a negative correlation.

(B) and (C) Application of LASSO regression, and the multivariate logistic regression, respectively, in analysis of the distinct infiltrates of immune cells in IPF lung tissue and control tissue.

(D) Significantly differential expression of genes of interest in IPF lung tissue and control tissue, as determined with the Wilcoxon test; ^*^p < 0.05; ^**^p< 0.01; ^***^p < 0.001.


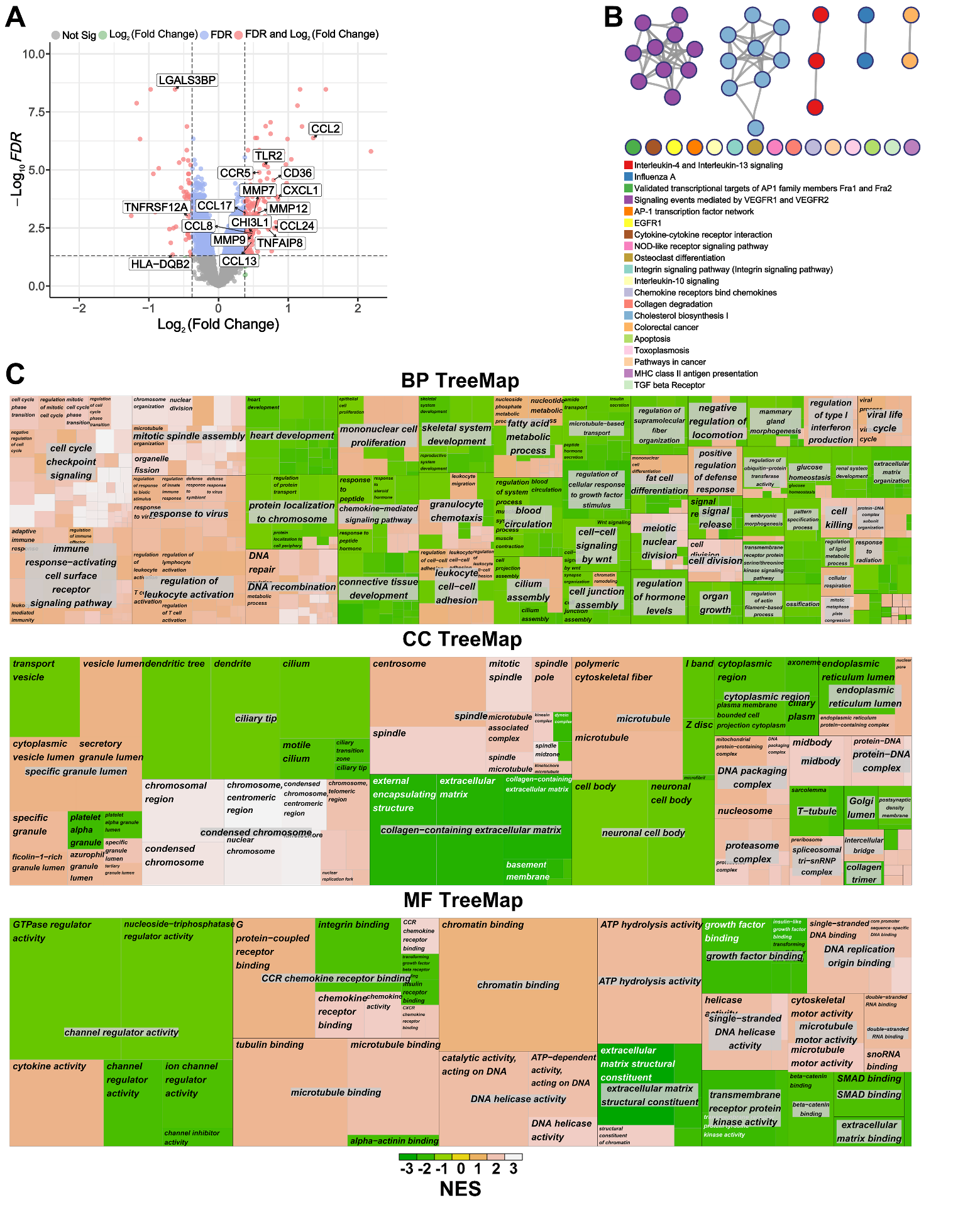


**Figure S2.** **Bioinformatics analysis of alveolar macrophages in idiopathic pulmonary fibrosis.**

(A) Volcano plot of differentially expressed genes (DEGs) in IPF alveolar macrophages compared with control alveolar macrophages.

(B) Enrichment analysis of gene expression, determined with CTpathway.

(C) Treemap of GSEA analysis in IPF alveolar macrophages compared with control alveolar macrophages, determined with gseGO.


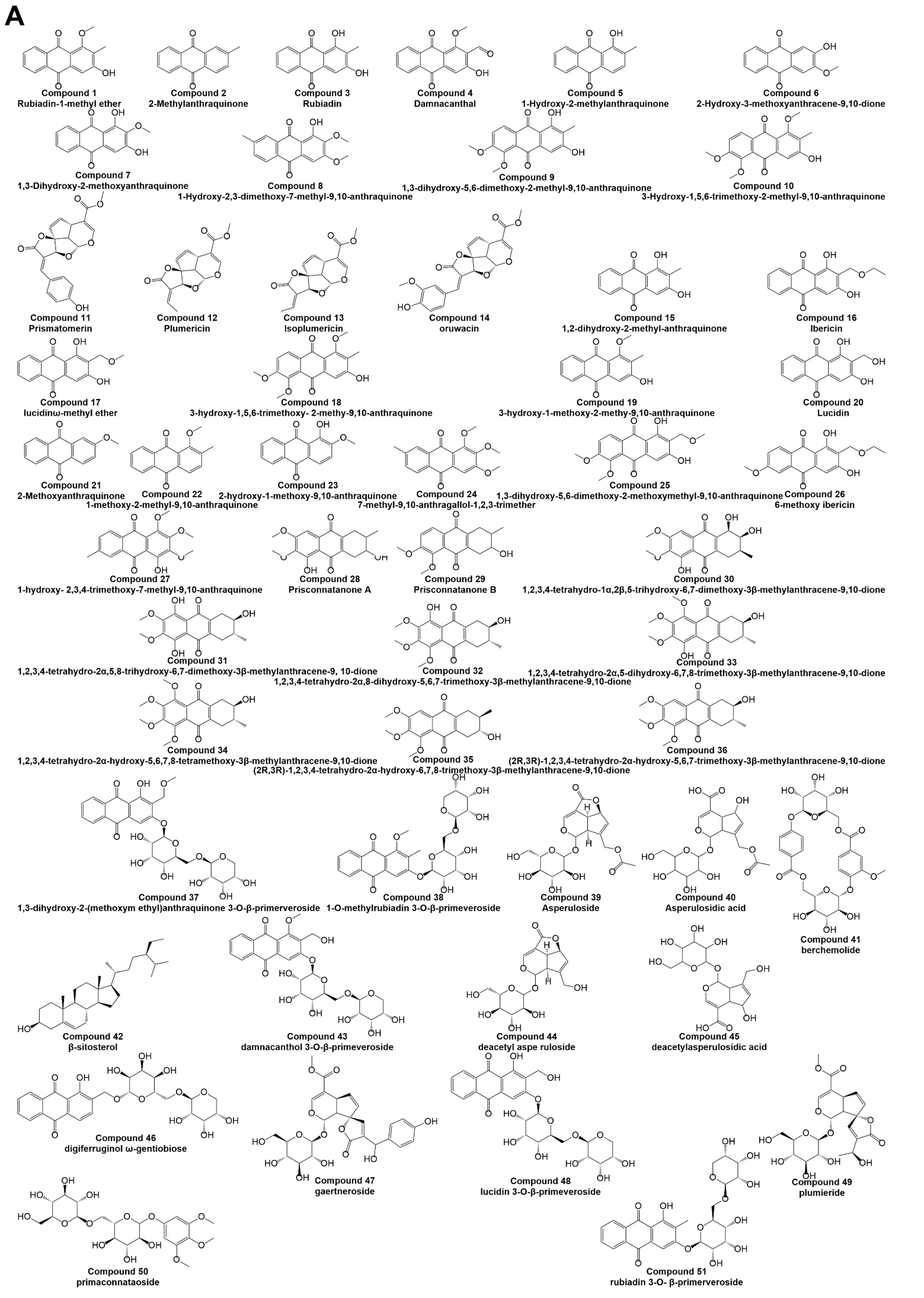


**Figure S3. Network pharmacology analysis reveals potential therapeutic targets and pathways of *Prismatomeris connata* Y. Z. Ruan root in idiopathic pulmonary fibrosis.**

(A) Fifty-one compounds with known structures found in the root of *Prismatomeris connata* Y. Z. Ruan.


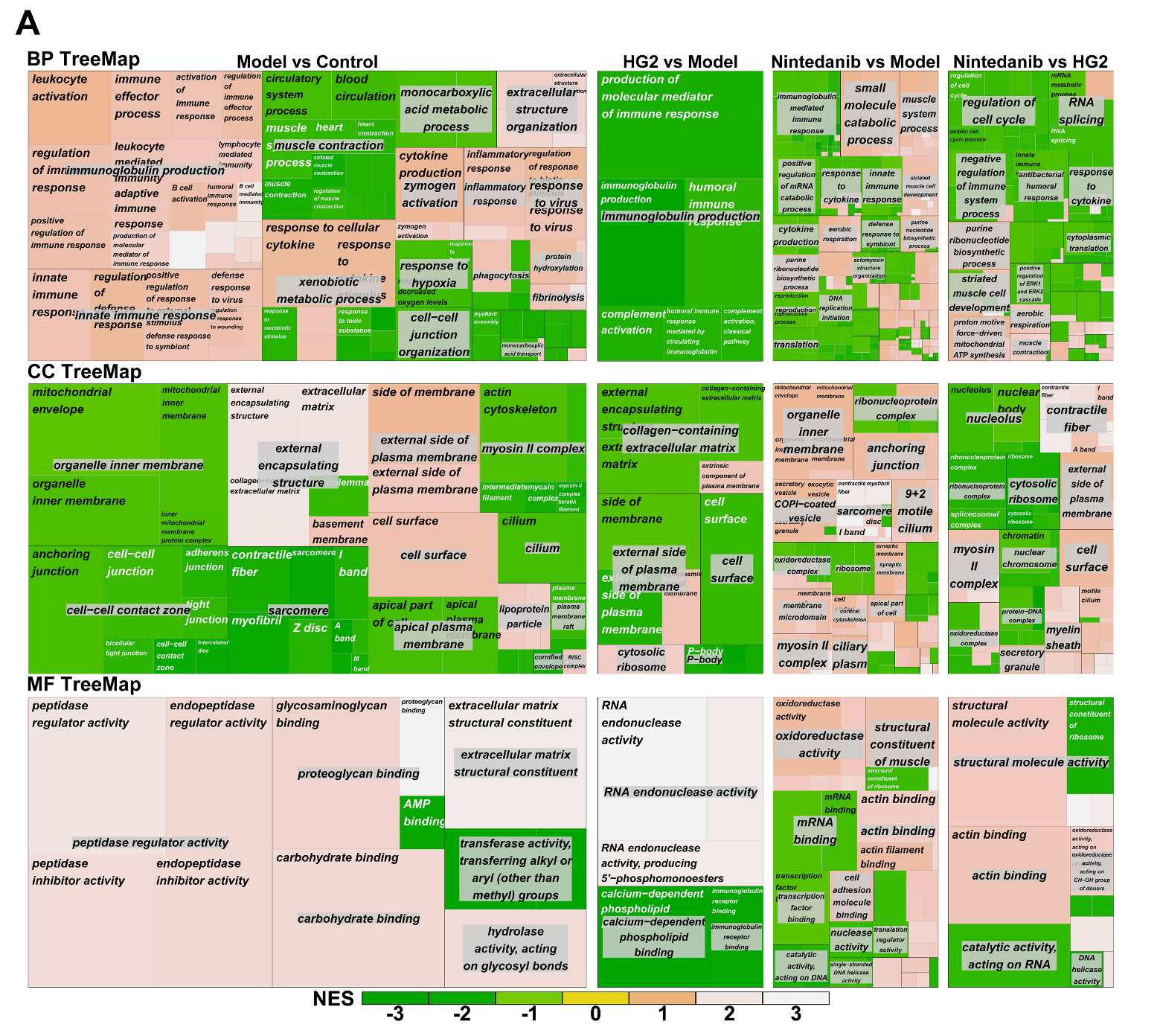


**Figure S4. Proteomic strategies uncover the potential pathogenesis of idiopathic pulmonary fibrosis and the mechanism of HG2.**

(A) Treemap of GSEA analysis in BLM-induced lung fibrosis tissue compared with control tissue, determined with gseGO.


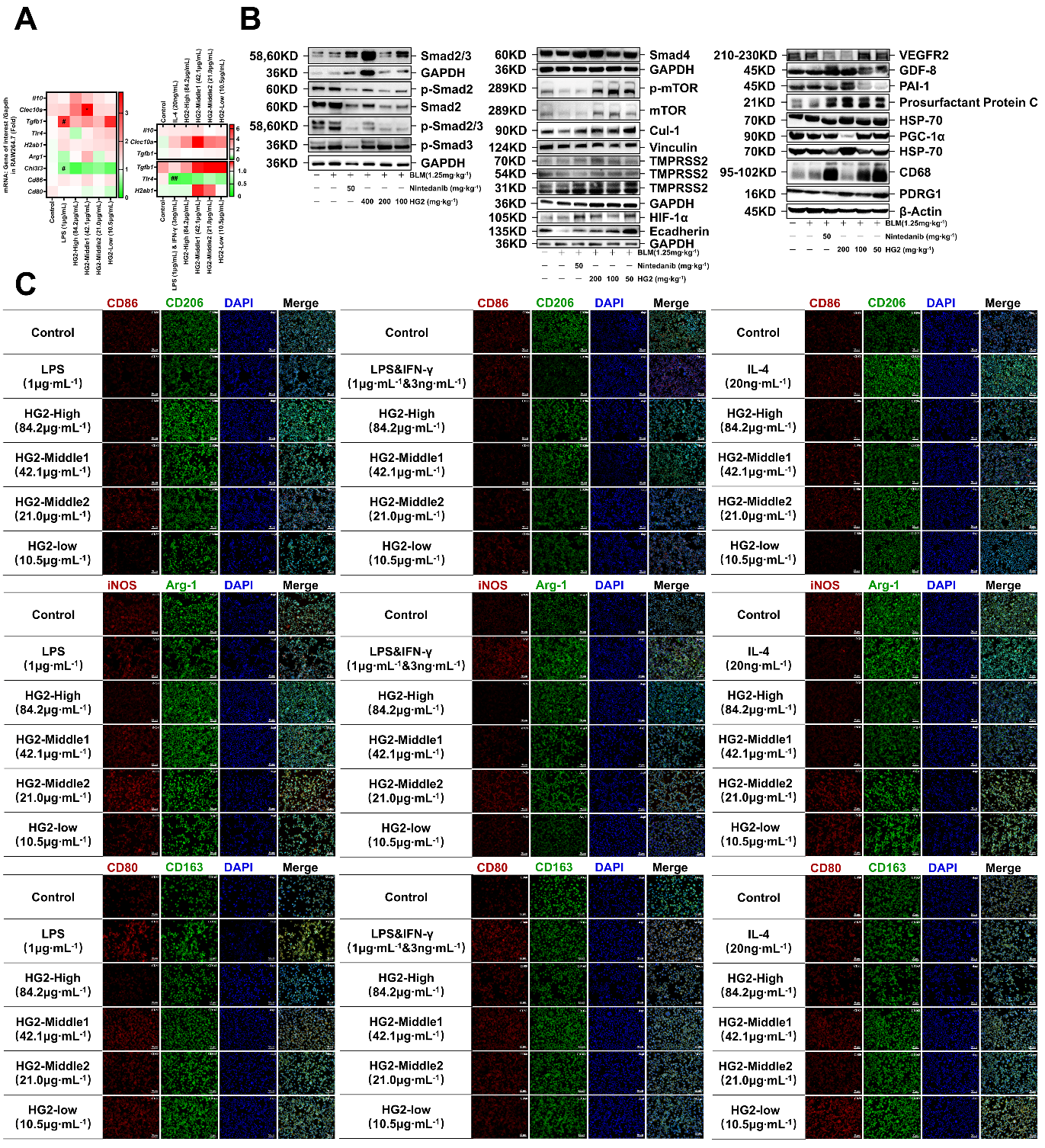


**Figure S5. HG2 modulates the expression of proteins in the TGF-β/Smad pathway in BLM-induced pulmonary fibrosis mice and macrophage polarization in vitro.**

(A) The mRNA expression of polarization-related markers in RAW264.7 cells was assessed by qPCR following stimulation with LPS (1μg/mL) /LPS (1μg/mL) combined with IFN-γ (3ng/mL) /IL-4 (20ng/mL), and intervention with HG2 (n=3). Compared with the control group, ^###^p<0.001, ^##^p<0.01, ^#^p<0.05. Compared with the model group, ^*^p<0.05, ^**^p<0.01, ^***^p<0.001.

(B) After 14 days of bleomycin and HG2 administration at different doses, the expression of related proteins in the lung tissue of mice was determined by Western Blot (n=3). Statistical values were expressed as mean ±SD.

(C) Immunocytochemistry was employed to assess the expression of CD206, CD86, iNOS, Arg-1, CD80, and CD163 in RAW264.7 cells stimulated by LPS (1μg/mL) / LPS (1μg/mL) combined with IFN- γ (3ng/mL) / IL-4 (20ng/mL). After 12 hours of inoculation, the cells were conditionally stimulated for an additional 12 hours, followed by immunocytochemical staining. Scale: 50μm.


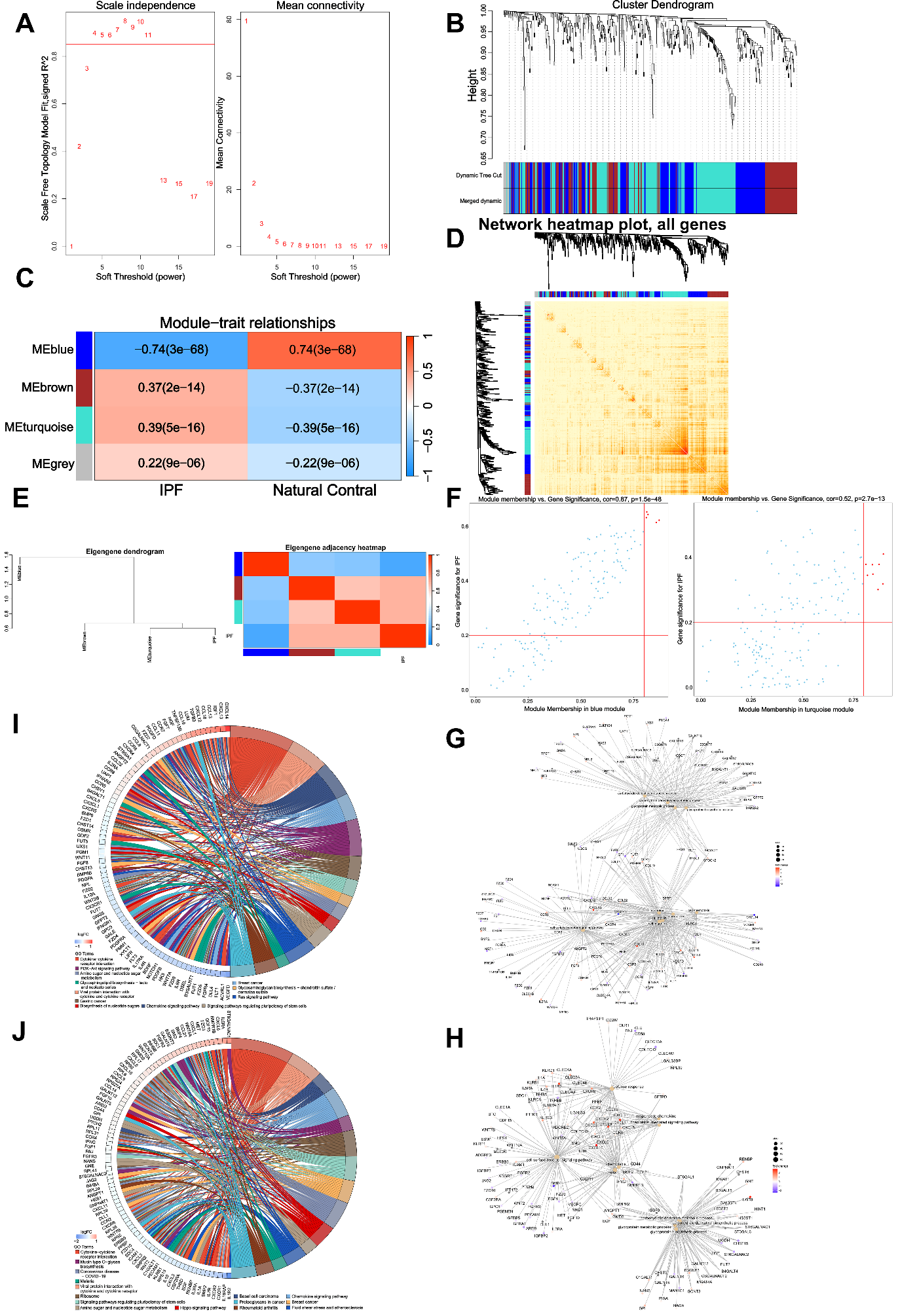


**Figure S6. Weighted gene co-expression network analysis between IPF alveolar macrophages and control alveolar macrophages.**


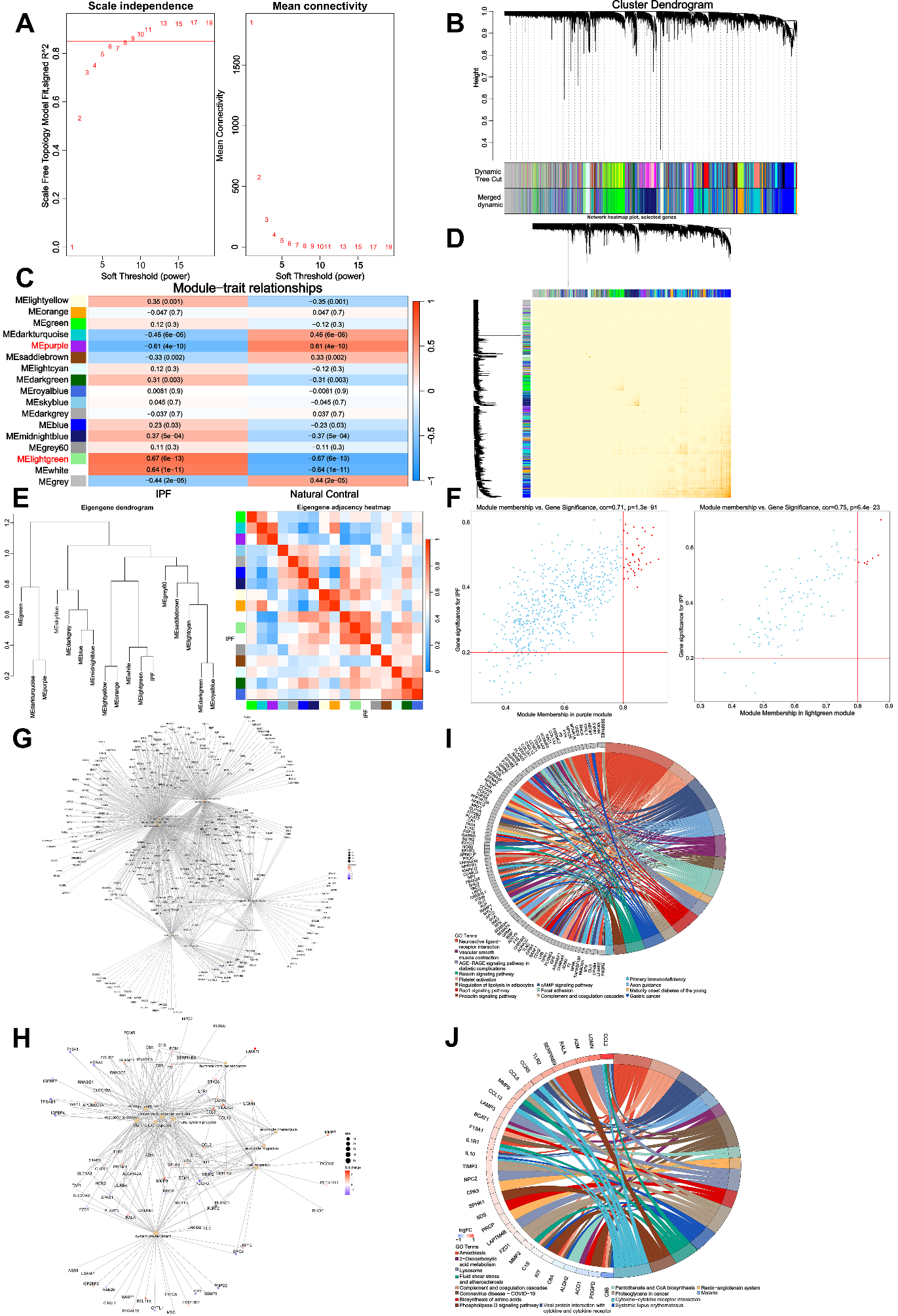


**Figure S7. Weighted gene co-expression network analysis between IPF lung tissue and control lung tissue.**
